# Innovative research of xian-Ling-Gu-bao herbal residue in regulating soluble carbon-to-nitrogen ratio to promote the growth and nutritional quality of *Pleurotus ostreatus*: A metabolomics and gut microbiota perspective

**DOI:** 10.1016/j.fochx.2025.103467

**Published:** 2025-12-29

**Authors:** Zefen Zhu, Huixin Li, Zhengfang Qi, Qin Cen, Xuefeng Zeng, Yichun Sun

**Affiliations:** a School of Liquor and Food Engineering, College of Life Sciences, Guizhou Provincial Key Laboratory of Agricultural and Animal Products Storage and Processing, Guizhou Provincial Key Laboratory of New Quality Processing and Storage of Ecological Specialty Food, School of Resources and Environmental Engineering, Guizhou Provincial Key Laboratory of Animal Genetics, Breeding and Reproduction in the Plateau Mountainous Region, Ministry of Education, Guizhou University, Guiyang 550000, China; b Sinopharm Group Tongjitang (Guizhou) Pharmaceutical Co, Ltd, Guiyang 550000, China

**Keywords:** Agricultural waste, Yield, Bioactives, Polysaccharide, Enzyme activity, Protein

## Abstract

This study evaluated the cultivation of *Pleurotus ostreatus* on a composite substrate consisting of Xin-Ling-Gu-Bao herbal residue (XLGB) and sauce-flavor Baijiu lees (SFL), both agro-industrial by-products rich in lignocellulose and bioactive compounds. The soluble carbon-to‑nitrogen ratio (SC/N) of the substrate showed a strong positive correlation with fungal growth (*r* = 0.89, *p* < 0.001). A formulation containing 50 % XLGB and 50 % SFL significantly enhanced lignocellulolytic enzyme activity, producing 520.28 g of mushrooms across three flushes, resulting in a biological efficiency of 148.65 %. This was 14.12 % and 43.78 % higher than the efficiency achieved with the control and traditional substrates, respectively. This substrate also facilitated the transfer of flavonoids and phenolics into the fruiting bodies and increased the abundance of beneficial bacteria during *in vitro* fermentation. Overall, these findings demonstrate an efficient, low-cost cultivation strategy for *Pleurotus ostreatus* and highlight the high-value, eco-friendly utilization of agro-industrial residues.

## Highlights

• Investigated the Impact of SC/N on the Growth of *P. ostreatus* (*r* = 0.89, *p* < 0.001).
• Optimized Substrate Formulation for Enhanced *P. ostreatus* Growth (B.E. = 148.65 %).
• Successfully Increased the Nutrition and Bioactive Compounds of *P. ostreatus*.
• Achieved the Resource Utilization of Agricultural By-products.
• The 50 %XLGB group of *P. ostreatus* had a positive impact on the gut microbiota.

## Graphical abstract

**Figure:**
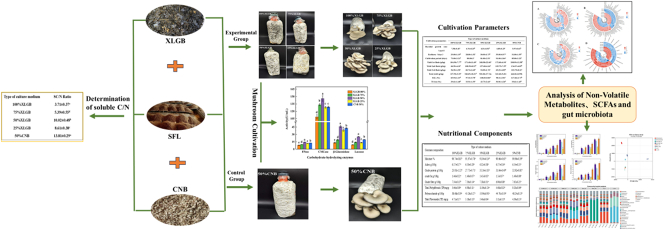

## 1 Introduction

Xian-Ling-Gu-Bao herbal residue (XLGB) is a byproduct derived from the extraction process of Xian-Ling- Gu-Bao capsules, a leading traditional Chinese medicine for osteoporosis treatment in China (Li et al., 2021). The raw material, composed of six herbs—*Epimedium*, *Dipsacus*, *Psoralea*, *Salvia miltiorrhiza*, *Rehmannia*, and *Anemarrhena*—meets the quality standards outlined in the Chinese Pharmacopoeia, ensuring the safety and consistency of the starting materials (Chen et al., 2016). Approximately 9000 tons of herbal residues are discarded annually as byproducts of Xian-Ling-Gu-Bao capsule production. Fresh XLGB herbal residue retains substantial amounts of lignin, cellulose, flavonoids, polyphenols, alkaloids, and other bioactive compounds (Pan et al., 2023). However, current disposal methods—such as biochemical fermentation, composting, and incineration—are inefficient in degrading lignocellulosic components and suffer from high costs, low added value, and environmental pollution (Chen et al., 2024). Thus, developing cost-effective and eco-friendly strategies for the valorization of traditional Chinese medicine residue remains a critical challenge for the pharmaceutical industry (Chen et al., 2024). To date, few studies have explored the high-value utilization of XLGB residues, underscoring the significance of this research.

*Pleurotus ostreatus*, one of the most popular edible fungi globally, accounts for 21 % of the world's mushroom supply (Li et al., 2025). It is highly valued for its rich nutritional composition (*e.g.*, proteins, carbohydrates) and bioactive compounds (*e.g.*, terpenoids, phenolics), which confer antioxidant, anti-inflammatory, and antitumor properties. As a white-rot fungus. *Pleurotus ostreatus* efficiently degrades lignin, cellulose, and hemicellulose in conventional substrates like cottonseed hulls and sawdust *via* secreted enzymes (Xu et al., 2013). However, rising cultivation costs have emerged due to declining cottonseed hull availability and stricter environmental policies limiting wood harvesting. Notably, Xian-ling-Gu-bao herbal residue (XLGB) is priced at 14 USD/ton—20 times lower than cottonseed hulls (285 USD/ton). Moreover, the uniform use of traditional substrates (*e.g.*, cottonseed hulls, sawdust, corncobs) across China's mushroom industry has hindered breakthroughs in regional *Pleurotus ostreatus* quality.

Studies indicate that carbon-to‑nitrogen (C/N) balance is critical for mycelial growth and fruiting body development (Economou et al., 2017). XLGB, with its abundant lignin and cellulose, could serve as a low-cost, stable carbon source, potentially addressing the industry's urgent need for affordable substrates while improving mushroom quality. Nevertheless, challenges such as nitrogen deficiency and limited flavor-enhancing compounds in XLGB-based cultivation require further investigation.

In previous studies, our team found that distiller's grains from sauce-flavor Baijiu production, as a byproduct of the brewing industry, contain abundant proteins that can provide nitrogen sources for the growth of *Pleurotus ostreatus*. Moreover, the bioactive polyphenols and distinctive flavor compounds from the distiller's grains were successfully transferred into the fruiting bodies of *Pleurotus ostreatus* (Zhou et al., 2023). Additionally, our team's preliminary research demonstrated that varying ratios of soluble carbon to nitrogen (SC/N) differentially affect the growth of *Pleurotus ostreatus*, which aligns with the findings of (Deshaware et al., 2021) regarding the optimal carbon and nitrogen sources for mycelial growth of Cantharellus cibarius. Soluble carbon and nitrogen sources are more readily absorbed by edible fungi, promoting more vigorous mycelial growth.

As reported by Gupta & Jana et al., (2017), the growth of edible fungal mycelia primarily relies on soluble nutrients, while extracellular enzymes are secreted to degrade insoluble lignins and celluloses into absorbable forms. The carbon-to‑nitrogen (C/N) ratio in the cultivation substrate is a critical factor influencing the growth of edible fungi (Gbolagade et al., 2006). However, there have been few reports on the initial SC/N ratio in cultivation substrates for *Pleurotus ostreatus*.

Therefore, this study utilizes XLGB and sauce-flavor Baijiu lees as carbon (SFL) and nitrogen sources for *Pleurotus ostreatus* cultivation, further investigating the effects of the initial SC/N ratio of the cultivation substrate on mushroom growth, as well as the feasibility and optimal formulation for cultivating *Pleurotus ostreatus* with XLGB and SFL. Additionally, ultra-high performance liquid chromatography-mass spectrometry (UHPLC-MS)-based untargeted metabolomics was employed to detect and identify whether bioactive compounds such as terpenoids, phenolics, and flavonoids were transferred to the fruiting bodies. The impact of the cultivated *Pleurotus ostreatus* fruiting bodies on human gut health, specifically focusing on short-chain fatty acids (SCFAs) and gut microbiota composition, was evaluated through *in vitro* fermentation.

This research aims to provide new theoretical foundations for the selection of cultivation materials in the edible mushroom industry, enhance the utilization rate and economic value of XLGB and SFL, reduce the cultivation cost of *Pleurotus ostreatus*, improve its nutritional value, leverage the biotechnological value of waste byproducts such as herbal residues and winery lees, mitigate environmental pollution, and offer a novel solution for the green and high-value utilization of agricultural and industrial waste.

## 2 Materials and methods

### 2.1 Materials

The *Pleurotus ostreatus* strain was provided by Xishui Edible Fungi Research Institute (Guizhou, China). Fresh Xian-ling-Gu-bao herbal residues (XLGB) were obtained from Sinopharm Group Tongjitang (Guizhou) Pharmaceutical Co., Ltd. (Guizhou, China), sauce-flavor Baijiu lees (SFL) from Guizhou Guijiu Group Co., Ltd. (Guizhou, China), and corncobs (CNB) from Chengli Fungi Co., Ltd. (Hubei, China). Chemical reagents, including PDB medium, agar, yeast extract powder (Shanghai Biowish Biotechnology Co., Ltd.), potassium phosphate, sodium bicarbonate, sodium chloride (Tianjin Komiou Chemical Reagent Co., Ltd.), lignin and cellulose assay kits, Folin-Ciocalteu reagent, rutin standard, and potassium acetate (Shanghai Macklin Biochemical Co., Ltd.) were commercially sourced.

### 2.2 Cultivation of *Pleurotus ostreatus*

#### 2.2.1 Spawn preparation

Corn grains were soaked overnight, boiled for 1 h, drained, and adjusted to pH 6–7 with 2 % lime. After bottling and sterilization (121 °C, 1.5 h), *Pleurotus ostreatus* slant cultures were inoculated aseptically and incubated at 25 °C in darkness for 15 days until fully colonized.

#### 2.2.2 Evaluation of growth parameters

Four substrate formulations were prepared: 100 % XLGB, 75 % XLGB +25 % SFL, 50 % XLGB +50 % SFL, and 25 % XLGB +75 % SFL. A control group (50 % corncob +50 % SFL) was included following (Zhou et al., 2023). Each formulation was adjusted to pH 8–9 with 2 % lime and 65 % moisture content. Substrates (1 kg per bag) were packed into polypropylene bags (17 × 15 × 24 cm; 20 bags per formulation), sterilized (121 °C, 1.5 h), and inoculated with *Pleurotus ostreatus* spawn (10 %, *w*/w) under aseptic conditions.

Fungus bags were incubated at 25 °C until full colonization (mycelial growth distance recorded). For fruiting induction, fungus bags were opened and transferred to a cultivation room (90 % RH, periodic misting, and aeration). The following parameters were recorded: primordia formation time, yield over three flushes, earliness index, biological efficiency, and dry matter loss. Harvested mushrooms were freeze-dried (48 h), ground (100-mesh sieve), and stored in desiccators for further analysis.

### 2.3 Determination of substrate materials SC/N, Total phenol, and flavonoid content

#### 2.3.1 Determination of SC/N

The SC/N of the five substrate formulations was determined using a distilled water extraction method (Yang et al., 2021). Each sample (10 g dry weight) was placed in a beaker containing 1000 mL of distilled water and extracted for 12 h, with three blank control groups set up. After extraction, the solution was filtered through a 0.45 μm membrane. The soluble carbon content was determined using a TOC analyzer, and the soluble nitrogen content was measured using the alkaline potassium persulfate ultraviolet spectrophotometric method. The SC/N ratio of each sample was then calculated.

#### 2.3.2 Determination of total phenol (TP) and flavonoid (TF) content

Modified from previous studies (Bajalan et al., 2016) 0.5 g of dry-weight and pulverized XLGB, SFL, and CNB were added to 50 mL centrifuge tubes, with three parallel samples per group. Distilled water without any sample addition was used as the blank control. Each tube was supplemented with 30 mL of 70 % ethanol solution and subjected to ultrasonic extraction for 90 min, followed by centrifugation (4 °C, 8000 rpm). The supernatant was collected as the sample for analysis. The total flavonoid and total phenol contents of the samples were measured, and the total flavonoid and total phenol contents in each cultivation medium were calculated.

The method for determining total flavonoid content was modified from previous studies (Bajalan et al., 2016). The sample solution (1 mL) was quantitatively transferred to a 10 mL volumetric flask and treated with equal volumes of potassium acetate (1 mol/L) and aluminum nitrate (0.1 mol/L) solutions. Following dilution to the mark with deionized water and vortex mixing, the reaction mixture was allowed to stand for 1 h prior to spectroscopic measurement at 420 nm. The standard curve was constructed using rutin solutions (25 - 100 μg/m L). The total flavonoid contents were expressed as mg of rutin equivalents (RE) per gram of a sample (mg RE/g).

The total phenol content was determined using the Folin-Ciocalteu reagent method (Bajalan et al., 2016). 1 mL of the sample solution was added to a 25 mL volumetric flask, followed by 6 mL of deionized water and 1 mL of Folin-Phenol reagent (1.0 mol/L). After shaking well and allowing it to stand for 6 min, 4 mL of sodium carbonate solution (10.6 %, *w*/*v*) was added. The mixture was allowed to stand for 60 min, then the volume was adjusted to the mark with deionized water. The absorbance was measured at 760 nm. The standard curve was constructed using gallic acid solutions (20-100 μg/m L). The total phenolic contents were expressed as mg of gallic acid equivalents (GAE) per gram of a sample (mg GAE/g).

### 2.4 Determination of enzyme activity

According to Edema et al. (2024) with minor modifications. After full colonization of *Pleurotus ostreatus* mycelia in the substrate, 3 g samples were homogenized in 30 mL sodium acetate buffer (0.1 M, pH 4.8) and shaken (120 rpm, 25 °C, 2 h). The supernatant was collected by centrifugation (5000 rpm, 4 °C) as crude enzyme extract.

Laccase (ABTS method): A mixture of 30 μ L enzyme extract, 100 μL sodium acetate buffer (0.1 M, pH 3), and 70 μL ABTS (2 mM) was incubated for 3 min. Absorbance at 420 nm was measured, with one unit (U/mL) defined as the amount of enzyme oxidizing 1 nmol ABTS per min per g sample.

Filter Paperase (FPA), Carboxymethyl Cellulase (CMCase), and β-Glucosidase (DNS method):

FPA: 0.5 mL enzyme extract +1 mL buffer + Whatman No. 1 filter paper strip (1 × 6 cm), incubated at 50 °C (60 min). CMCase: 0.5 mL enzyme extract +1 mL 1 % CMC—Na, incubated at 50 °C (30 min). β-Glucosidase: 0.5 mL enzyme extract +1 mL 1 % salicin, incubated at 50 °C (30 min).

Reactions were terminated with 1.5 mL DNS, boiled (5 min), and diluted to 10 m L. Absorbance at 540 nm was measured. One unit (U/mL) equaled 1 μ mol glucose released per min.

### 2.5 Variations in cellulose and lignin concentrations in growth medium

The content of cellulose and lignin in the culture medium of each group of raw materials and after harvesting the third substrate was determined. Slightly modified from the method of (Li et al., 2022), appropriate amounts of culture medium (randomly selected from the packages) were taken separately, dried and crushed through a 40-mesh sieve, and the samples were retained for measurement. The lignin content of the samples was determined by Lignin Content Assay Kit (Solarbio China), and the cellulose content of the samples was determined by Cellulose Content Assay Kit (Macklin China).

### 2.6 Determination of basic nutritional components in *Pleurotus ostreatus*

Modified from Zhou et al. (2023),the fresh sample moisture content was determined using a moisture analyzer (SFY-30, Guan Ya Technology Co., Ltd., Shenzhen, China). Crude protein content was analyzed by the Kjeldahl method, total fat by Soxhlet extraction, ash content by combustion at 550 °C in a muffle furnace, and total fiber content by acid hydrolysis.

### 2.7 Determination of major bioactive compounds in *Pleurotus ostreatus*

#### 2.7.1 Preparation of extracts

1.0 g of *Pleurotus ostreatus* powder was accurately weighed into a 50 mL amber centrifuge tube, mixed with 30 mL of 70 % ethanol, and ultrasonically extracted for 90 min (Rosyida et al., 2024). The supernatant was collected after centrifugation (9000r, 5 min) for analysis.

Total flavonoid and phenolic contents in fruiting bodies were determined following the method described in Section 2.3.2.

#### 2.7.2 Determination of crude polysaccharide

The determination of crude polysaccharide content was performed according to the method described by Rosyida et al. (2024), with slight modifications. Briefly, 1.00 g of powdered *Pleurotus ostreatus* was accurately weighed into a 250 mL round-bottom flask, followed by the addition of 50 mL distilled water and 15 mL concentrated hydrochloric acid. The mixture was hydrolyzed under reflux condensation at 100 °C for 3 h with magnetic stirring. After cooling to room temperature, the hydrolysate was filtered and thoroughly washed, then diluted to 250 mL with distilled water to obtain the test solution. The polysaccharide content in fruiting bodies was determined by the phenol‑sulfuric acid method using a standard curve established with glucose solutions at different concentrations.

### 2.8 Determination of non-volatile metabolites in *Pleurotus ostreatus*(LC-MS)

Following the analysis results, the flat mushroom substrates from the 50 % XLGB group, exhibiting the highest yield and mycelial extracellular enzyme activity, were chosen for further investigation alongside the 50 % CNB (control group). Subsequently, the non-volatile metabolites of the substrates from both groups were identified using LC-MS.

Sample preparation: Aliquots (50 ± 5 mg) of finely powdered fungal fruiting bodies were accurately weighed into 2 mL microcentrifuge tubes containing a 6-mm stainless-steel grinding bead. Methanolic extraction was performed by adding 400 μL of 80 % (*v*/v) aqueous methanol, followed by mechanical disruption in a cryogenic homogenizer (6 min, 50 Hz, −10 °C). Subsequent ultrasonic-assisted extraction was conducted under controlled conditions (30 min, 40 kHz, 5 °C). To facilitate phase separation, samples were incubated at −20 °C for 30 min and then centrifuged (13,000 ×*g*, 4 °C, 15 min). The resulting supernatant was carefully collected and transferred to LC-MS-compatible vials equipped with micro-inserts. For analytical quality assurance, a pooled quality control (QC) sample was prepared by combining 20 μL aliquots from each individual extract.

LC-MS conditions were adapted from Yang et al. (2021) with minor modifications: Chromatographic separation was performed on an ACQUITY UPLC HSS T3 column (100 mm × 2.1 mm i.d., 1.8 μ m, Waters, Milford, USA) maintained at 40 °C. The mobile phase consisted of (A) 95 % water+5 % acetonitrile with 0.1 % formic acid and (B) 47.5 % acetonitrile+47.5 % isopropanol+5 % water with 0.1 % formic acid. The injection volume was 3 μL. Mass spectrometric detection was performed in both positive and negative ionization modes with a mass range of *m*/*z* 70–1050. The ion spray voltages were set at 3500 V (positive) and − 3000 V (negative), with sheath gas at 50 arb, auxiliary gas at 13 arb, and ion source temperature at 450 °C. A collision energy of 20–40-60 V was applied in cycles.

### 2.9 *In vitro* fermentation by human gut bacteria

Fresh fecal samples were collected from four healthy volunteers (2 males and 2 females) with no antibiotic use in the past 6 months or probiotic consumption within 2 weeks prior. The collection of human fecal specimens was conducted in compliance with the ethical guidelines authorized by the Medical Ethics Committee of Guizhou University (Approval No. HMEE-GZU-2025-T016). After obtaining informed consent, samples were immediately transferred to an anaerobic environment. Homogenized feces were diluted 1:10 (*w*/*v*) in PBS (0.01 M, pH = 7.4), vortexed, filtered through four-layer gauze, and centrifuged (5000r, 5 min). The supernatant was collected as the inoculum.

The basal medium was prepared as described by prior research (Tan et al., 2024), containing (per L): 2.0 g yeast extract, 2.0 g peptone, 0.02 g hemin, 0.5 g bile salts, 0.5 g L-cysteine, 2.0 g NaHCO3, 0.1 g NaCl, 0.04 g KH_2_PO_4_, 0.04 g K2HPO4, 0.01 g CaCl2·6H2O, 0.01 g MgSO4·7H2O, 0.01 g vitamin K1, 0.01 g resazurin, and 2 mL Tween 80. Test substrates (50 % XLGB, 50 % CNB, inulin) were dissolved in the medium (10 mg/mL) and sterilized (121 °C, 20 min), with inulin (IN) as positive control and no carbohydrate source as negative control (Blank). Fermentation was initiated by inoculating 9 mL medium with 1 mL fecal suspension in triplicate, followed by anaerobic incubation at 37 °C for 0, 12, 24, and 36 h. Samples were stored at −80 °C until analysis.

#### 2.9.1 Determination of SCFAs

The fermented samples were centrifuged (8000 rpm, 15 min), and 2 mL of the supernatant was mixed with 0.2 mL of 25 % (*w*/*v*) metaphosphoric acid. After standing for 2 h at room temperature, the mixture was filtered through a 0.22 μ m membrane for GC analysis.

Modified from Tan et al. (2024).Short-chain fatty acids (SCFAs) were quantified using an Agilent 7890 A gas chromatograph equipped with a flame ionization detector (FID) and a DB-FFAP capillary column (30 m × 250 μ m × 0.25 μ m). Helium was used as the carrier gas (2.0 mL/min, split ratio 10,1). The oven temperature program was as follows: initial hold at 90 °C for 3 min, ramped to 170 °C at 15 °C/min, then to 220 °C at 20 °C/min (held for 5 min). The injection volume was 1 μ L. Hydrogen and air flows for the FID were maintained at 40 mL/min and 400 mL/min, respectively.

#### 2.9.2 DNA extraction and 16S rRNA gene sequencing

Genomic DNA of the microbial community was isolated from the pelleted fraction of the fermented culture medium employing the E.Z.N.A.® Soil DNA extraction system (Omega Bio-tek, Norcross, GA, U.S.) following the supplier's recommended procedures. The integrity of the extracted DNA was assessed through electrophoretic separation on a 1 % agarose matrix, with quantitative and qualitative evaluations performed using a Nano-Drop 2000 UV–Vis spectrophotometric analyzer (Thermo Scientific, Wilmington, DE).

The hypervariable V3-V4 segments of bacterial 16S ribosomal RNA genes were polymerase chain reaction (PCR)-amplified employing barcode-indexed universal primers 338F (5’-ACTCCTACGGGAGGCAGCAG-3′) and 806R (5’-GGACTACHVGGGTWTCTAAT-3′). Amplification reactions were carried out in a thermal cycler (T100, Bio-Rad Laboratories, Hercules, CA) with the following thermal profile: primary denaturation at 95 °C (3 min); 27 amplification cycles comprising denaturation (95 °C, 30 s), primer annealing (55 °C, 30 s), and elongation (72 °C, 30 s); concluding with terminal elongation (72 °C, 10 min) before cooling to 4 °C. The 20 μ L reaction volume consisted of: 4 μ L 5× Trans Start Fast Pfu reaction buffer, 2 μ L deoxynucleotide triphosphate mix (2.5 mM), 0.8 μ L each of forward and reverse primers (5 μ M), 0.4 μ L Trans-Start Fast-Pfu DNA polymerase, and 10 ng genomic DNA template.

Amplification products were resolved electrophoretic ally on 2 % agarose gels and subsequently purified using a commercial PCR product purification system (Yuhua Biological Technology, China). Quantification of purified amplicons was performed using a Qubit 4.0 fluorometric quantification system (Thermo Fisher Scientific, Waltham, MA). Sequencing library construction was executed with the NEXTFLEX Rapid DNA-Seq Kit through sequential steps including: (1) adapter ligation; (2) magnetic bead-mediated elimination of adapter dimer artifacts; (3) PCR-mediated library amplification; and (4) final library purification using magnetic bead-based size selection. Paired-end sequencing was subsequently conducted on an Illumina sequencing platform (PE250/PE300 chemistry, Illumina, Inc., San Diego, CA).

### 2.10 Statistical analysis

Triplicate measurements were obtained for all experimental conditions. Quantitative data were processed in Excel 2019 and reported as means ± SD. Statistical comparisons were conducted in SPSS 21.0 (IBM, USA), with significance defined at *p* < 0.05. Figures were generated using Origin 2024 (Origin Lab, USA) and the Majorbio Cloud Platform (https://www.majorbio.com/).

## 3 Results and discussion

### 3.1 Analysis of substrate materials SC/N, total phenol, and flavonoid content

#### 3.1.1 Analysis of initial SC/N

The SC/N results for the five groups of substrates are shown in Table 1. The SC/N values for the 100 % XLGB, 75 % XLGB, 50 % XLGB, 25 % XLGB, and 50 % CNB groups were 3.71 ± 0.37, 5.39 ± 0.55, 10.02 ± 0.48, 8.61 ± 0.38, and 13.81 ± 0.29, respectively, with significant differences (*p* < 0.05). An appropriate C/N ratio is a key factor for edible fungi growth (Yu et al., 2021). In traditional cultivation, the C/N ratio of cultivation materials such as sawdust, corn cobs, and cottonseed hulls is often roughly considered, without taking into account the bioavailability of complex carbon sources such as lignin and cellulose, or the carbon and nitrogen utilization mechanisms of edible fungi.

**Table 1:** Analysis of soluble carbon-to‑nitrogen ratio(SC/N Ratio), total phenol, and total flavonoids in substrate raw materials. XLGB: Xian-ling-Gu-bao herbal residues; SFL: sauce-flavor Baijiu lees; CNB: corncob; 100 %XLGB: 100 % XLGB,75 %XLGB: 75 % XLGB +25 % SFL,50 %XLGB: 50 % XLGB +50 % SFL,25 %XLGB: 25 % XLGB +75 % SFL,50 %CNB: 50 % CNB + 50 % SFL (control group). ND:Not determined. Different letters in the same column indicate significant differences (*p* < 0.05) in cultivation parameters among different substrate formulations.

	Type of culture medium							
	100 %XLGB	75 %XLGB	50 %XLGB	25 %XLGB	50 %CNB		SFL	CNB
SC/N Ratio	3.71 ± 0.37^e^	5.39 ± 0.55^d^	10.02 ± 0.48^b^	8.61 ± 0.38^c^	13.81 ± 0.29^a^		ND	ND
TP (mg GAE/g)	3.22 ± 0.02^b^	ND	ND	ND	ND		5.28 ± 0.14^a^	3.01 ± 0.13^c^
TF (mg RE/g)	11.26 ± 1.67^a^	ND	ND	ND	ND		7.46 ± 0.41^b^	1.18 ± 0.46^c^

Edible fungi decompose organic compounds such as lignin and cellulose in the cultivation material into soluble small molecules through the secretion of extracellular enzymes, thereby acquiring nutrients. Studies have shown that soluble carbon and nitrogen sources are more readily absorbed by mycelium, promoting the secretion of extracellular enzymes and accelerating substrate degradation (Papaspyridi et al., 2011). Therefore, variations in the initial SC/N ratio may affect the absorption of carbon and nitrogen sources by the mycelium, which in turn influences its growth and colonization on the substrate. Subsequent research indicated that the initial SC/N ratio was positively correlated with the yield of *Pleurotus ostreatus* (*r* = 0.89, *p* < 0.001). Thus, investigating the SC/N ratio may provide a theoretical basis for selecting cultivation materials for edible fungi, though the optimal range and mechanisms still require further optimization and validation.

#### 3.1.2 Analysis of total phenol and flavonoid content

Evaluating the characteristic active components of cultivation materials is crucial for understanding the bioactive composition of edible mushroom fruiting bodies. Flavonoids and polyphenols are the major active components in XLGB and SFL. Table 1 shows the total flavonoid and total phenol content in XLGB, SFL, and CNB. The results indicate that the remaining total flavonoid content in XLGB, SFL, and CNB are 11.26 mg/g, 7.46 mg/g, and 1.18 mg/g, respectively, while the total phenol content is 3.22 ± 0.02 mg/g, 5.28 ± 0.14 mg/g, and 3.01 ± 0.13 mg/g, respectively. The flavonoid and phenol contents in XLGB and SFL are significantly higher than those in CNB (*p* < 0.05), with the flavonoid content in XLGB being 11 times higher than that in CNB. This suggests that both XLGB medicinal residue and SFL contain abundant active components that have not been fully utilized. It is worth noting that these tests use different chemical components and reference compounds, which may result in the total flavonoid content being higher than the total phenolic content. Previous studies have shown that the characteristic components of edible fungi are closely related to their cultivation substrates. (Karataş, 2021) found that using different agricultural waste materials as substrates for *Pleurotus ostreatus* cultivation can increase the corresponding characteristic component content in the fruiting bodies, and this is positively correlated. Therefore, we hypothesize that the cultivation substrates used in this study promote an increase in the corresponding active component content in *Pleurotus ostreatus* fruiting bodies.

### 3.2 Analysis of enzyme activity

**Fig. 1:**
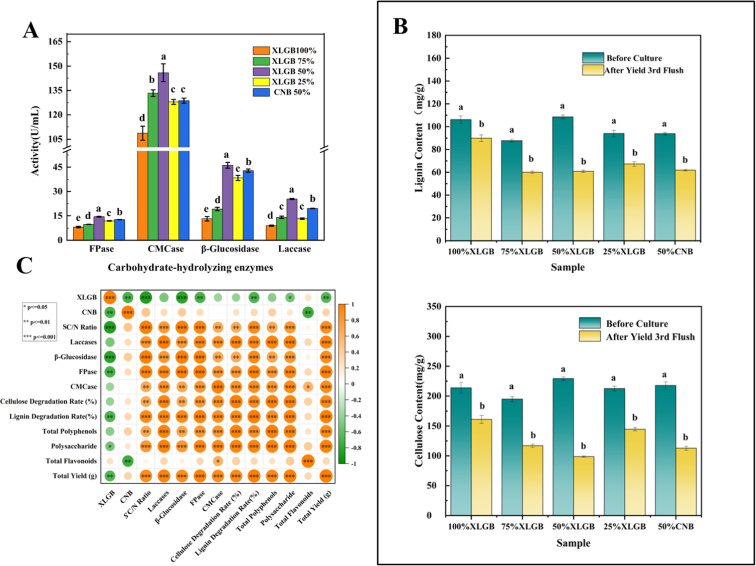
A: Enzyme activity Analysis of five group. B: Variations in cellulose and lignin concentrations in growth medium of five group. Different letters represent significant differences between samples (*p* < 0.05). C: The correlation analysis.100 %XLGB:100 %XLGB+0 %SFL;75 %XLGB:75 %XLGB+25 %SFL;50 %XLGB: 50 %XLGB +50 %SFL;25 %XLGB: 25 %XLGB +75 %SFL.

The activity of lignocellulolytic enzymes is closely associated with the yield and quality of *Pleurotus ostreatus*. In this study, the activities of four enzyme were measured across five substrate groups. As shown in Fig. 1 A, the 50 % XLGB group exhibited optimal performance, with significantly higher activities of filter paperase (14.48 U/mL), carboxymethyl cellulase (145.92 U/mL), β-Glucosidase (46.17 U/mL), and laccase (25.35 U/mL) compared to the control group (*p* < 0.05). Specifically, carboxymethyl cellulase, filter paperase, and laccase activities increased by 13.37 %, 14.06 %, and 29.55 %, respectively. These results indicate more vigorous mycelial growth in the 50 % XLGB group, which is consistent with previous reports demonstrating a positive correlation between fungal extracellular enzyme activity and mycelial biomass (Economou et al., 2017). As a white-rot fungus, *Pleurotus ostreatus* primarily absorbs soluble carbon and nitrogen sources but cannot directly utilize solid substrates. Instead, it secretes extracellular enzymes to degrade macromolecules into soluble compounds.We hypothesize that the initial SC/N ratio (8–9) in the 50 % XLGB group may facilitate nutrient absorption and mycelial colonization, thereby influencing extracellular enzyme activity. Correlation analysis (Fig. 1C) revealed positive relationships between SC/N ratio and filter paperase (*r* = 0.95), carboxymethyl cellulase (*r* = 0.67), β-Glucosidase (*r* = 0.99), and laccase (*r* = 0.80). Pedraza-Zapata et al. (2017) further demonstrated that variations in carbon and nitrogen concentrations affect mycelial development *via* oxidase pathways. Enhanced lignocellulolytic enzyme activity promotes substrate degradation, thereby providing essential nutrients for subsequent growth and fruiting body formation. To further validate these findings, we recorded and analyzed growth parameters, including yield and dry matter loss, across the experimental groups.

### 3.3 Analysis of growth parameters

The cultivation performance across experimental groups is summarized in (Table 2). The 50 % XLGB group achieved a total yield of 520.28 g (three flushes) with a biological efficiency of 148.65 %, representing 14.12 %, 43.78 %, and 18.35 % increases compared to 50 % CNB (446.81 g), traditional corncob, and cottonseed hull substrates, respectively (Yang et al., 2013; Zhou et al., 2023). These results demonstrate the superior suitability of the 50 % XLGB substrate for *Pleurotus ostreatus* cultivation. Notably, the 50 % XLGB group exhibited 1.3-fold higher dry matter loss than the control, indicating enhanced mycelial utilization of carbon and nitrogen compounds. This phenomenon may be attributed to the optimized initial the SC/N ratio (8–9) in the 50 % XLGB substrate (Fig. 1 A), which facilitated nutrient assimilation and colonization, thereby stimulating extracellular enzyme production and subsequent lignocellulose degradation. The strong positive correlation between SC/N and yield (*r* = 0.89, *p* < 0.001; Fig. 1C) further supports this mechanism. Our findings align with (Schiesser et al., 1989), who reported that *Pleurotus ostreatus* preferentially consumes soluble intercellular compounds before degrading structural lignocellulose polymers. The 50 % XLGB +50 % SFL mixture emerges as an optimal cultivation substrate, with initial SC/N identified as a key growth-determining factor that could complement traditional C/N ratio criteria for substrate selection. However, dynamic changes in SC/N during cultivation require further investigation.

**Table 2:** Cultivation parameters of *Pleurotus ostreatus* grown on substrates composed of fresh Xian-ling-Gu-bao herbal residues (XLGB) mixed with sauce-flavor Baijiu lees (SFL), corncob (CNB) mixed with sauce-flavor Baijiu lees, and their combinations. 100 %XLGB: 100 % XLGB,75 %XLGB: 75 % XLGB +25 % SFL,50 %XLGB: 50 % XLGB +50 % SFL,25 %XLGB: 25 % XLGB +75 % SFL,50 %CNB: 50 % CNB + 50 % SFL (control group). Different letters in the same column indicate significant differences (*p* < 0.05) in cultivation parameters among different substrate formulations.

Cultivation parameters	Type of culture medium				
	100 %XLGB	75 %XLGB	50 %XLGB	25 %XLGB	50 %CNB
Mycelial growth rate(cm/d)	7.58 ± 0.41^a^	6.76 ± 0.25^b^	6.51 ± 0.51^b^	4.85 ± 0.25^c^	5.97 ± 0.61^b^
Earliness(days)	25.00 ± 1.15^d^	28.00 ± 1.52^c^	30.00 ± 1.73^b^	35.00 ± 0.57^a^	31.00 ± 1.53^b^
Cultivation period (days)	73.00 ± 3.21^d^	80.00 ± 2^c^	81.00 ± 2.51^c^	91.00 ± 2.64^a^	85.00 ± 3.21^b^
Yield 1st flush (g/kg)	104.50 ± 7.77^d^	171.40 ± 11.49^c^	268.58 ± 22.06^a^	172.40 ± 4.94^c^	208.95 ± 12.38^b^
Yield 2nd flush (g/kg)	66.50 ± 4.10^e^	108.50 ± 8.33^d^	157.60 ± 6.16^a^	105.75 ± 7.55^c^	134.07 ± 4.85^b^
Yield 3rd flush (g/kg)	56.50 ± 1.50^c^	60.74 ± 2.64^b^	94.09 ± 1.76^a^	65.25 ± 4.05^b^	103.79 ± 8.11^a^
Total yield (g/kg)	227.50 ± 3.33^e^	340.65 ± 18.21^d^	520.28 ± 27.74a	343.40 ± 9.45c	446.81 ± 8.59b
B.E. (%)	65.94 ± 1.23^d^	97.32 ± 5.78^c^	148.65 ± 8.81^a^	98.11 ± 3.01^c^	127.66 ± 2.73^b^
TS lost (%)	29.61 ± 1.48^d^	33.51 ± 1.53^c^	43.73 ± 2.64^a^	30.56 ± 1.26^d^	35.61 ± 1.43^b^

### 3.4 Variations in cellulose and lignin concentrations in growth medium

Cellulose and lignin serve as the primary energy sources for *Pleurotus ostreatus* growth, with their degradation directly affecting substrate utilization efficiency and fruiting body formation. As shown in (Fig. 1B), following the third harvest, the 50 % XLGB group exhibited significantly higher degradation rates for cellulose (57 %) and lignin (43.24 %) than other groups, representing 1.27 times and 1.19 times increases over the 50 % CNB group, respectively (*p* < 0.05). The enhanced substrate degradation and nutrient utilization were correlated with increased fruiting body yield and reduced dry matter content (Table 2), which is consistent with the established positive correlation between the degradation capacity of white-rot fungi and enzyme activity. The 50 % XLGB group demonstrated superior lignocellulolytic enzyme activity, resulting in more efficient substrate decomposition. Excessive residual lignin and cellulose negatively impact subsequent fruiting body development and yield. These results confirm that the 50 % XLGB +50 % SFL mixture represents an optimal cultivation substrate. Our findings demonstrate *Pleurotus ostreatus*' exceptional degradation capability for XLGB and SFL, enhancing their biotechnological value and economic potential while reducing environmental pollution. This approach provides a viable solution for high-value utilization of agro-industrial waste. However, further analysis of the mushroom's nutritional quality is warranted.

### 3.5 Analysis of basic nutritional components in *Pleurotus ostreatus*

The results of nutrient composition of *Pleurotus ostreatus* fruiting bodies in each group are presented in (Table 3). Protein content is a key nutritional indicator. Among groups with different proportions of XLGB, the 50 % XLGB treatment exhibited the highest protein level (35.16 g/100 g), representing a 31.97 % increase than the 100 % XLGB group. This demonstrates that SFL can provide nitrogen sources for fungal growth. Previous studies confirm that nitrogen-rich substrate optimization enhances protein synthesis in *Pleurotus ostreatus* (Gupta et al., 2013). However, protein content decreased in the 25 % XLGB group (equivalent to >75 % SFL), likely due to reduced extracellular enzyme activity, which impaired nitrogen assimilation and protein biosynthesis. Xiao et al. (2017) reported that protein variation may correlate with fungal adaptation to lignocellulosic substrates, consistent with our extracellular enzyme activity data (fig. 1 A). Therefore, our study demonstrates that XLGB serves as an effective nutritional substrate for *Pleurotus ostreatus* cultivation, not only enhancing the mushroom's nutritional quality but also improving the biotechnological and economic value of both XLGB and SFL byproducts.

**Table 3:** Nutrient and major bioactive compound levels in *Pleurotus ostreatus* grown on different substrate formulations. Moisture: Water content per 100 g of fresh mushrooms; Others: Content in corresponding dried mushrooms. Different letters in the same column indicate significant differences (*p* < 0.05) in nutrient composition among fruiting bodies cultivated on different substrates.

composition	Type of culture medium				
	100 %XLGB	75 %XLGB	50 %XLGB	25 %XLGB	50 %CNB
Moisture %	90.74 ± 0.81^**b**^	91.97 ± 0.78^**a**^	91.04 ± 0.14^**b**^	90.66 ± 0.61^**b**^	90.96 ± 0.39^**b**^
Ashes g/100 g	6.17 ± 0.57^**a**^	6.50 ± 0.29^**a**^	6.32 ± 0.38^**a**^	6.57 ± 0.36^**a**^	6.54 ± 0.35^**a**^
Crude protein g/100 g	23.92 ± 1.52^**d**^	27.75 ± 0.71^**c**^	35.16 ± 1.05^**a**^	31.64 ± 0.49^**b**^	32.92 ± 0.81^**b**^
crude fat g/100 g	1.46 ± 0.32^**a**^	1.49 ± 0.07^**a**^	1.45 ± 0.05^**a**^	1.5 ± 0.07^**a**^	1.49 ± 0.08^**a**^
Crude fiber g/100 g	7.34 ± 0.13^**b**^	7.36 ± 0.14^**a**^	7.58 ± 0.31^**a**^	6.96 ± 0.08^**c**^	7.65 ± 0.25^**a**^
TP (mg GAE/g)	3.66 ± 0.09^**e**^	6.98 ± 0.11^**c**^	11.06 ± 0.24^**a**^	4.68 ± 0.02^**d**^	9.20 ± 0.06^**b**^
Polysaccharide g/100 g	38.68 ± 0.39^**e**^	45.26 ± 0.27^**c**^	53.96 ± 0.91^**a**^	44.70 ± 0.50^**d**^	48.24 ± 0.13^**b**^
TF mg RE/g	4.71 ± 0.17^**c**^	5.18 ± 0.15^**b**^	5.46 ± 0.04^**a**^	5.11 ± 0.13^**b**^	4.39 ± 0.15^**d**^

### 3.6 Analysis of major bioactive components in *Pleurotus ostreatus*

*Pleurotus ostreatus* is a medicinally significant edible fungus, valued for its rich bioactive compounds, including polyphenols, polysaccharides, and flavonoids.

Our experimental results (Table 3) demonstrate that the 50 % XLGB group exhibited significantly higher phytochemical contents in the fruiting body of *Pleurotus ostreatus* compared to other groups, with total phenols (11.06 mg/g), flavonoids (5.46 mg/g), and polysaccharide (53.96 g/100 g) showing increases of 20.21 %, 24.37 %, and 11.86 %, respectively, relative to the control group (*p* < 0.05). On the one hand, this difference may be attributed to the fact that the residual polyphenols and flavonoids in the XLGB substrate (Table 1) were absorbed and transformed by the *Pleurotus ostreatus*, and that the XLGB and SFL were enriched in polyphenols and flavonoids, whereas the CNB contained lower levels of such components (Table 1). On the other hand, the activity of lignocellulolytic enzymes secreted by the mycelium is also one of the key factors affecting the accumulation of active ingredients in *Pleurotus ostreatus*. During fermentation, lignin-degrading enzymes (*e.g.*, laccases) cleave ether bonds between phenolic compounds and lignin, as well as ester bonds linking phenolics to carbohydrates or proteins, thereby releasing free phenolic substances (Zhang et al., 2024). Lan et al. (2023) further demonstrated that ligninolytic enzymes participate in flavonoid biosynthesis, explaining the elevated flavonoid content in the 50 % XLGB group. Enhanced lignocellulolytic activity also promotes hydrolysis of cellulose β-1,4-glycosidic bonds, generating glucose as a carbon source for polysaccharide synthesis. Additionally, phenolic compounds have been reported to stimulate β-glucan production, which may further contribute to the higher polysaccharide content in the 50 % XLGB group. Our findings reveal that the 50 % XLGB +50 % SFL mixture is an optimal substrate for *Pleurotus ostreatus* cultivation, as it enhances lignocellulolytic enzyme activity and facilitates precursor mobilization. However, the specific biosynthetic pathways and regulatory mechanisms underlying bioactive compound accumulation require further investigation to refine cultivation strategies and maximize the value of *Pleurotus ostreatus*, XLGB, and SFL.

### 3.7 Correlation analysis

To further investigate the interactions between the initial SC/N ratio of the substrate and extracellular enzyme activity, yield, and bioactive compound accumulation in *Pleurotus ostreatus*, Pearson correlation analysis was performed (fig. 1C). The results demonstrated that the activities of four extracellular enzymes (laccase, β-glucosidase, filter paperase, and carboxymethyl cellulase) were significantly positively correlated with the initial SC/N ratio. Among them, laccase, β-glucosidase, and filter paperase exhibited highly significant correlations with SC/N (*r* = 0.80, 0.99, and 0.95, respectively; *p* < 0.001), while carboxymethyl cellulase showed a slightly weaker correlation (*r* = 0.67, *p* < 0.01), possibly due to substrate specificity. These findings indicate that SC/N is a key factor regulating extracellular enzyme secretion in *Pleurotus ostreatus*.

Moreover, SC/N and extracellular enzyme activity were positively correlated with fruiting body yield (*r* = 0.89, *p* < 0.001). An optimal SC/N ratio may enhance mycelial metabolism, promoting the synthesis of lignocellulose-degrading enzymes. The decomposition of lignocellulose in the substrate releases soluble carbon sources, providing direct energy for fruiting body development and thereby increasing yield (Bánfi et al., 2015). Additionally, residual phenolic compounds in XLGB and SFL may be released by laccase activity, absorbed by the mycelium, and accumulated in the fruiting bodies. The increased β-glucosidase activity likely facilitates polysaccharide precursor supply, further enhancing polysaccharide accumulation in the fruiting bodies. Our study found that the extracellular enzyme activity and yield of mycelium were optimal in the 50 % XLGB group. Therefore, the amount of XLGB added should take into account the characteristics of the composite materials. In the experimental groups where SFL was used as the nitrogen source, the addition of XLGB showed a statistically significant negative correlation with SC/N ratio and extracellular enzyme activity. It is important to note that in future studies on the relationship between the SC/N ratio of raw materials and their proportions, the specific properties of the raw materials should be carefully considered.

This study reveals that the initial SC/N ratio of the substrate positively regulates the extracellular enzyme activity of oyster mushrooms, promoting the utilization efficiency of XLGB and SWL substrates, enhancing fruiting body yield, and driving the migration and transformation of bioactive substances. However, the SC/N range needs further optimization, and the molecular mechanisms of key enzymes involved in the synthesis of bioactive substances should be further elucidated.

### 3.8 Analysis of non-volatile metabolites in *Pleurotus ostreatus*(LC-MS)

According to the above results, we considered 50 % XLGB to be the optimal ratio. Therefore, we selected 50 % XLGB to further investigate the metabolic profile of *Pleurotus ostreatus* fruiting bodies, with 50 % CNB serving as the control group.

#### 3.8.1 Analysis of PCA

**Fig. 2:**
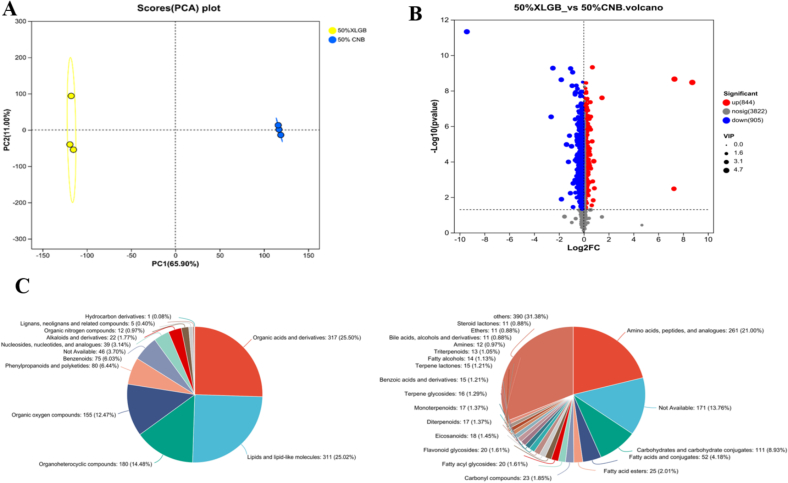
A: The total sample Principal component analysis (PCA) of 50 %XLGB and 50 %CNB samples. B: Volcano plot of the differential metabolites of 50 %XLGB *vs.* 50 %CNB·C: Classification of all the detected metabolites of 50 %XLGB and 50 %CNB samples.

Principal component analysis (PCA) revealed that the 50 % XLGB and 50 % CNB samples accounted for 76.90 % of the total variance (fig. 2 A), with PC1 (65.90 %) and PC2 (11.00 %). The high clustering of samples within each group along PC1 indicated good reproducibility, while the clear separation between the two groups along PC1 suggested significant differences in their metabolic profiles.

#### 3.8.2 Differential metabolite analysis

**Fig. 3:**
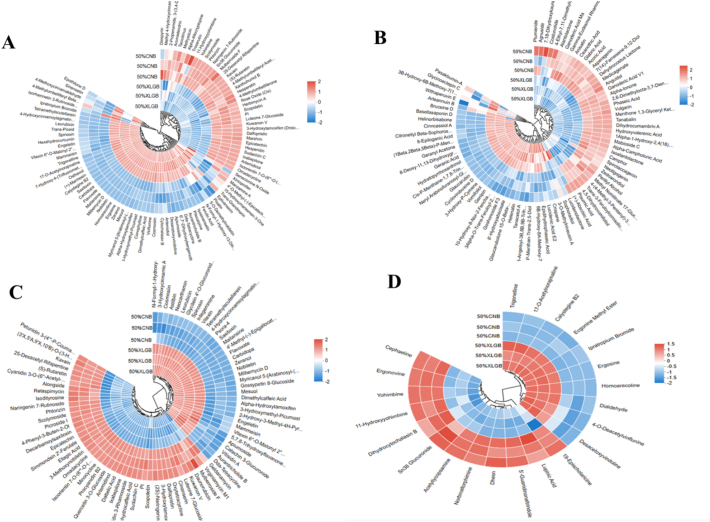
Heat map of clustering of four differential metabolites. A: amino acids; B: terpenoids; C: phenylpropanoids and polyketides; D: alkaloids.

Differential metabolites were preliminarily annotated by HMDB database matching after excluding indistinguishable isomers. Under the criteria of *P* < 0.05 and VIP > 1, a total of 1749 differential metabolites (including 844 upregulated) were identified (fig. 2B), covering alkaloids, flavonoids, and others, reflecting metabolic diversity between the two groups (fig. 2C). Further cluster heatmap analysis was performed on amino acids, phenylpropanoids and polyketides, terpenoids, and alkaloids (fig. 3).

As shown in (Fig. 3 A), among the top 100 relatively abundant amino acid metabolites, 68 % of the amino acid-related compounds in the fruiting bodies of the 50 % XLGB group exhibited significant upregulation, including L-ornithine, L-proline, N-linoleoyl-L-leucine, and 65 other amino acids and peptides. This result is in strong agreement with the previously observed increase in total protein content (Table 3), suggesting a direct relationship between XLGB supplementation and amino acid accumulation in the fruiting bodies.

This marked increase in amino acid levels in the 50 % XLGB group is likely due to the nutrient profile of the culture substrate, which contains nitrogen-rich compounds—such as proteins, amino acids, and nucleic acids—derived from medicinal plant tissues (roots, stems, and leaves) included in the XLGB formulation. During mycelial growth, these nitrogenous compounds are broken down by the enzymatic system of *Pleurotus ostreatus* (Bánfi et al., 2015), providing essential precursors for amino acid biosynthesis in the fruiting bodies.

In addition to the nutrient composition, the enhanced extracellular enzymatic activity observed in the 50 % XLGB group likely plays a central role in the observed increase in amino acids. The stimulation of lignocellulolytic enzymes facilitates the breakdown of the substrate, releasing more glucose (Bánfi et al., 2015). Glucose, in turn, serves as both an energy source for cellular processes—including active amino acid transport and biosynthesis—and a precursor for central metabolic intermediates (such as pyruvate, α-ketoglutarate, and oxaloacetate) (Yin et al., 2024), which act as key carbon skeletons for the synthesis of various amino acids.

Terpenoids, flavonoids, polyphenols, and alkaloids constitute the primary bioactive components of XLGB and contribute significantly to the medicinal value of edible fungi.

The results shown in (Fig. 3B) indicate that more than half of the detected terpenoids, including 8-Epiloganic acid, Goshonoside F3, Licoricesaponin E2, and 10-Formyltetrahydrofolate, were significantly upregulated. This may be attributed to the higher activity of the lignocellulolytic enzyme system in the 50 % XLGB group (Fig. 1), which promotes the degradation of lignocellulose and releases more glucose molecules as carbon sources. These glucose molecules can be metabolized to produce a large amount of acetyl-CoA (Halsey et al., 2017), thereby facilitating the biosynthesis of terpenoid compounds. In basidiomycetes, the formation of terpenoids requires sufficient supplies of both carbon and nitrogen sources (Yu et al., 2023). Furthermore, our study revealed that 8-Epiloganic acid present in the XLGB herbal residues could directly migrate into the fruiting bodies, contributing to the improved quality and commercial value of *Pleurotus ostreatus*.

The results shown in (Fig. 3C) indicate that a total of 15 flavonoid metabolites were significantly upregulated in the 50 % XLGB group among the 27 detected compounds, including pharmacologically active compounds such as flavoxate, engeletin, cosmosiin, and spinosin. These compounds have been reported to possess diverse bioactivities, including anti-inflammatory, antioxidant, and antitumor effects (Slámová et al., 2018). This increase may be attributed to the transformation and migration of active components from the culture substrate into the fruiting bodies under the action of enzymes. Flavonoids are major constituents of XLGB, and the characteristic metabolites of *Pleurotus ostreatus* are closely related to the composition of the culture substrate. Flavonoids present in the substrate can be released by lignin-degrading enzymes and taken up by fungal cells, where they are further modified by glycosyltransferases and methyltransferases. Such modifications increase their water solubility, facilitating storage in the cytoplasm and vacuoles (Slámová et al., 2018). The engeletin and cosmosiin detected in our study are glycosylated forms of flavonoids, which are likely absorbed directly or further modified after uptake.

In addition, phenolic and alkaloid compounds such as 4-Hydroxycinnamoylagmatine, purpurogallin, apiumoside, fraxetin, and ergosine were significantly upregulated in the 50 % XLGB group. Purpurogallin is a naturally occurring phenolic compound, and its increased content in the fruiting bodies of *Pleurotus ostreatus* may be attributed to the higher laccase activity in the 50 % XLGB group, as laccase can catalyze the conversion of pyrogallol to purpurogallin (Honda et al., 2017). Furthermore, the precursors of polyphenols and flavonoids are primarily derived from phenylalanine *via* the phenylpropanoid pathway (Zhang et al., 2025). The general upregulation of amino acid levels indicates that pathways related to aromatic amino acid metabolism were activated, which is conducive to the synthesis and accumulation of polyphenol and flavonoid secondary metabolites.

Overall, the enrichment and transformation of bioactive components such as flavonoids, polyphenols, and alkaloids in the fruiting bodies enhance their medicinal value, further supporting the suitability of XLGB herbal residues as a substrate for *Pleurotus ostreatus* cultivation. However, the effects of these mushrooms on the human gut remain unknown. Therefore, we conducted *in vitro* fermentation simulating the human gastrointestinal environment to further investigate the impact of the cultivated mushrooms on the gut microbiota.

### 3.9 *In vitro* fermentation by human gut bacteria

#### 3.9.1 Analysis of SCFAs

**Fig. 4:**
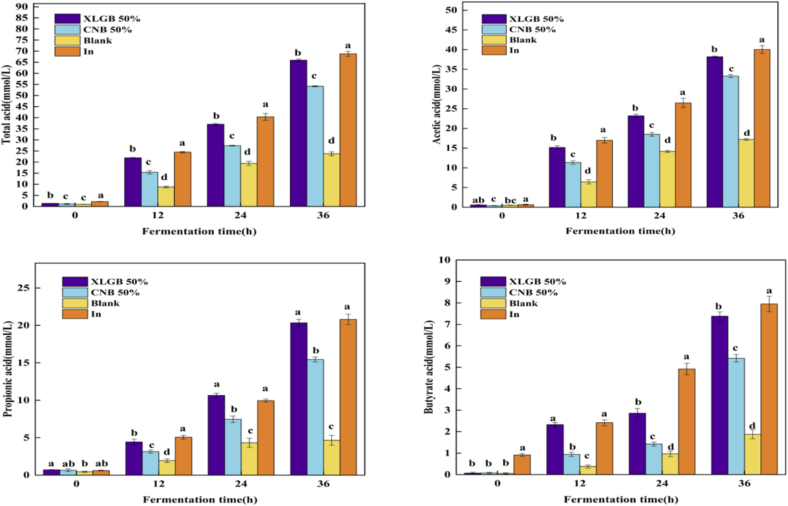
Powder of the fruiting body of *Pleurotus ostreatus* (50 %XLGB and 50 %CNB) were added as the fermentation substrates, and inulin (In) were used as a positive control, and the unadded carbon source (Blank) was used as a negative control group. Changes in SCFAs value during *in vitro* fermentation of different samples by human fecal microbiota.

SCFAs, particularly acetate, propionate, and butyrate (accounting for >90 % of total SCFAs) play crucial roles in modulating gut microbiota homeostasis and energy metabolism. After 36 h of *in vitro* fermentation, the 50 %XLGB, 50 %CNB, and In groups showed significantly higher SCFAs concentrations than the Blank group (fig. 4). The total SCFAs concentration in the 50 %XLGB group increased from 1.39 to 65.93 mmol/L, representing 2.7 times and 1.23 times increases compared to the Blank (23.74 mmol/L) and 50 %CNB (54.15 mmol/L) groups, respectively.

This pronounced accumulation of SCFAs can be attributed to a more vigorous and efficient microbial metabolism. The variation in total SCFAs may be linked to the polysaccharide content in fruiting bodies. Polysaccharides are key precursors in the synthesis of SCFAs, significantly increasing SCFAs levels in the feces of healthy hosts and improving gut microbiota metabolic function (Chen et al., 2024). As shown in (Table 3), the polysaccharide content in the 50 % XLGB group was significantly higher than in the 50 % CNB group. Additionally, the polyphenols and flavonoids enriched in the fruiting bodies may play a crucial prebiotic role in the human gut. Phenolic compounds selectively promote the growth and metabolic activity of beneficial bacteria, such as *Bifidobacterium* and *Bacteroides*, while natural flavonoid glycosides act as fermentation substrates in the colon, promoting SCFAs production(Liu et al., 2021). This may be the primary mechanism driving increased SCFAs production.

Acetate was the predominant SCFAs, with its concentration in the 50 %XLGB group rising from 0.61 to 38.19 mmol/L (122 % and 14.75 % higher than Blank and 50 %CNB groups, respectively). Moderate levels of acetic acid can provide energy to the gut and have potential anti-inflammatory and blood sugar-lowering effects (Chen et al., 2024). Propionate and butyrate exhibited similar trends, demonstrating a strong ability to regulate microbiota metabolism(Wu et al., 2021).

The trends in SCFAs levels after fermentation suggest that *Pleurotus ostreatus* can stimulate gut microbiota to produce SCFAs, thereby supporting gut health. The SCFAs content in the 50 % XLGB group was significantly higher than in the 50 % CNB (control) group, likely due to the higher levels of active compounds such as polysaccharides, flavonoids, and polyphenols in the 50 % XLGB group. These compounds help stabilize the gut microbiota, promote SCFAs production, and exhibit strong anti-inflammatory effects while maintaining gut barrier function (Hassan et al., 2021). Furthermore, SCFAs production is closely linked to gut microbiota activity, prompting further investigation into the impact of oyster mushrooms on gut microbiota.

#### 3.9.2 Composition, characteristics, and diversity of gut microbiota

**Fig. 5:**
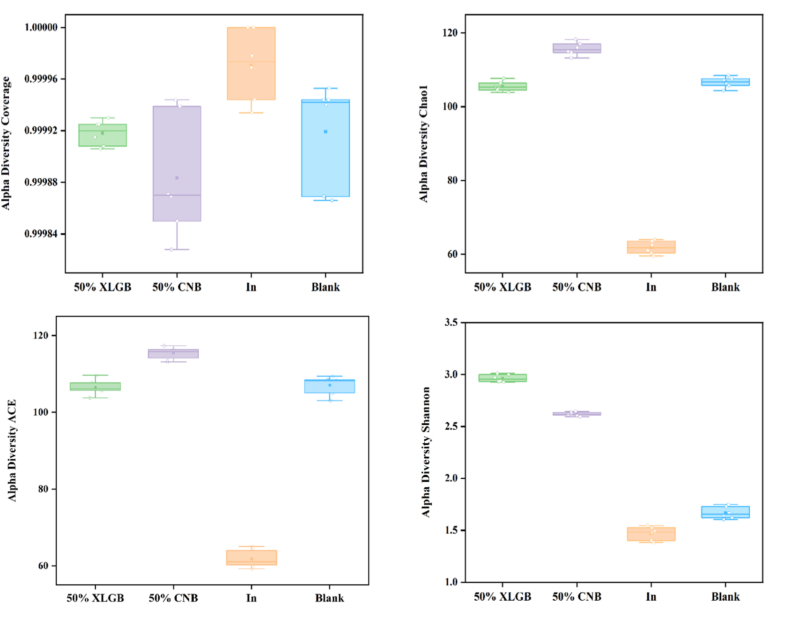
α-diversity analysis.

Coverage indices (>0.99) confirmed adequate sequencing depth. Following 36 h fermentation, both community richness (ACE and Chao indices) and diversity (Shannon index) decreased across all groups, with the most pronounced reduction observed in the In group, followed by the 50 %XLGB group (fig. 5). This decline may result from pH reduction caused by SCFAs production during carbohydrate fermentation, which likely inhibited certain pathogenic microorganisms—consistent with our SCFAs quantification results.

**Fig. 6:**
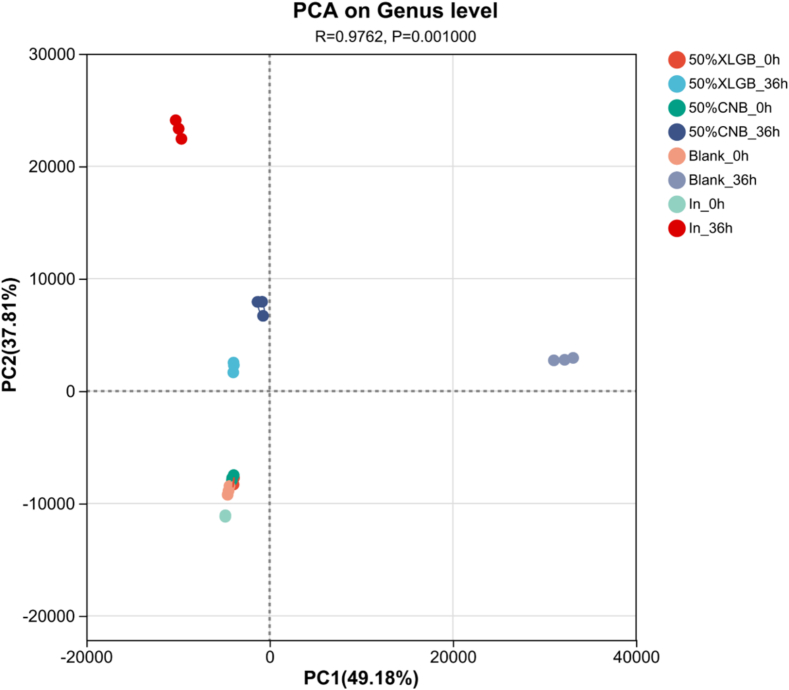
Analysis of β-diversity.

Principal component analysis (PCA) revealed distinct microbial community shifts (PC1: 49.18 %; PC2: 37.81 %; cumulative variance: 86.99 %;fig. 6). While initial microbiota compositions were similar (0 h), significant divergence occurred post-fermentation (36 h). Notably, the 50 %XLGB and 50 %CNB groups exhibited clear separation, indicating differential modulation of gut microbiota by their distinct compositional profiles.

**Fig. 7:**
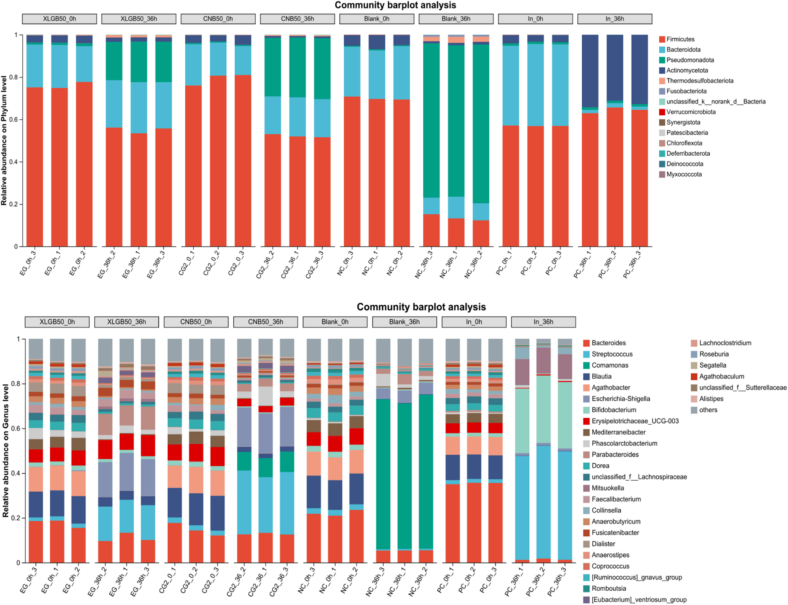
Relative abundance of bacterial communities at phyla level and at genus level.

To study the effects of two groups of *Pleurotus ostreatus* on the gut microbiota, the relative abundance of the top 30 gut microbes before and after fermentation at the phylum and genus levels was analyzed (Fig. 7). After 36 h of fermentation, the gut microbiota composition changed in all treatment groups. At the phylum level, the dominant phyla in all treatment groups were Firmicutes, Bacteroidota, Pseudomonadota, and Actinomycetota. Firmicutes can ferment polysaccharides and other carbohydrates in the gut microbiota to produce SCFAs, such as *Streptococcus* and *Mitsuokella*, which are commonly found in the human gut microbiota. Treptococcus is a typical gut microbe with a dual role. In moderation, *Streptococcus* ferments various carbohydrates to produce SCFAs and participates in energy metabolism. However, excessive growth of *Streptococcus* may be detrimental to host health, as an abnormal abundance of *Streptococcus* has been linked to diseases such as inflammatory bowel disease (IBD) and colorectal cancer (Chen et al., 2022). We observed that after 36 h of fermentation, the abundance of *Streptococcus* in the 50 % XLGB group increased 7-fold compared to before fermentation, although it remained lower than in the 50 % CNB group and the positive control. This may be due to the selective regulatory effect of polyphenols and terpenoids in the 50 % XLGB group on *Streptococcus*, helping to maintain gut microbiota homeostasis.

The relative abundance of *Mitsuokella* is positively correlated with butyrate levels in the gut. After 36 h of fermentation, the relative abundance of *Mitsuokella* in the 50 % XLGB group was higher than in the 50 % CNB and Blank groups, which is consistent with the results of butyrate content in each group (Fig. 4). Bacteroidota can ferment dietary cellulose as a carbon source for growth and produce SCFAs such as acetate and propionate, making it an important component of the human gut microbiota. After 36 h of fermentation, the relative abundance of *Bacteroidetes* in the 50 % XLGB and 50 % CNB groups increased to 22.36 % and 18.45 %, respectively, while the Blank group decreased to 10.21 %, In the 50 % XLGB and 50 % CNB groups, the relative abundance of *Parabacteroides* increased from 0.13 % to 9.48 % and 3.64 %, respectively. In contrast, we found that the relative abundance of *Parabacteroides* was very low in the Blank and In groups. *Parabacteroides* is a core member of the host gut microbiota, helping to occupy ecological niches and prevent pathogen colonization. Some studies consider *Parabacteroides* a potential next-generation probiotic due to its association with healthy metabolic states and its demonstrated potential to improve obesity and glucose homeostasis in animal models (Wu et al., 2025). This is a positive sign, as Bacteroidota, one of the most important polysaccharide-degrading microbial groups in the gut, showed a significant increase in overall abundance in the 50 % XLGB group. This suggests that the polysaccharides in the *Pleurotus ostreatus* from the 50 % XLGB group provide effective fermentation substrates for beneficial gut bacteria.

It is noteworthy that an excessively high ratio of Firmicutes to Bacteroidota has been linked to obesity and may negatively impact host health (Mao et al., 2025). And polyphenolic compounds have been shown to regulate the ratio of these two phyla (Mao et al., 2025), and our findings are consistent with this. This suggests that the polyphenols and other active compounds in the *Pleurotus ostreatus* from the 50 % XLGB group exert a specific prebiotic effect during fermentation, helping to modulate the gut microbiota in a beneficial way.

Pseudomonadota, commonly found in nature, especially *Pseudomonas aeruginosa*, is a human pathogen that may cause respiratory infections and inflammation when overgrown in the gut (Moule et al., 2024), After 36 h of fermentation, the relative abundance of Pseudomonadota in the Blank group increased to 72.89 %, while in the 50 % XLGB and 50 % CNB groups, the relative abundances were 18.09 % and 28.27 %, respectively. This suggests that *Pleurotus ostreatus* has an inhibitory effect on the growth of Pseudomonadota in the gut, possibly due to the higher content of polyphenols, flavonoids, terpenes, and other bioactive substances in the fruiting bodies of the 50 % XLGB group. Especially, polyphenols have been shown to selectively regulate gut microbiota, specifically increasing the relative abundance of beneficial bacteria while reducing harmful bacteria (Mao et al., 2025). This may be one of the key mechanisms through which consuming the *Pleurotus ostreatus* studied in our research could offer potential health benefits.

## 4 Conclusion

We systematically evaluated the feasibility of cultivating *Pleurotus ostreatus* using a mixture of XLGB and SFL. Our results highlight the key regulatory role of SC/N in determining *Pleurotus ostreatus* yield and quality. The 50 % XLGB +50 % SFL formula significantly increased the yield of *Pleurotus ostreatus* by 43.78 % compared to the traditional corncob substrate, while also enriching the fruiting bodies with active compounds such as polyphenols, flavonoids, and terpenoids, demonstrating the formula's advantage in enhancing nutritional and functional value. Further analysis indicated that an initial SC/N ratio of 8–9 promotes mycelial growth and colonization, suggesting that SC/N can serve as a useful theoretical parameter for screening and optimizing fungal cultivation substrates. However, the dynamic changes in SC/N during fruiting body development and its physiological effects still require further investigation. *In vitro* fermentation experiments showed that the mushrooms significantly promoted SCFAs and the proliferation of beneficial bacteria, demonstrating strong prebiotic potential. In conclusion, using XLGB as a cultivation substrate offers a cost-effective, efficient production pathway for the edible mushroom industry, while addressing environmental pollution and resource waste from herbal residue disposal. Future research should focus on the structural features, bioactivity, and synergistic mechanisms of *Pleurotus ostreatus* polysaccharides and polyphenols, and validate their physiological functions through animal models or human trials to advance the development and application of functional mushroom products.

## CRediT authorship contribution statement

**Zefen Zhu:** Writing – original draft, Visualization, Validation, Investigation. **Huixin Li:** Writing – review & editing, Supervision. **Zhengfang Qi:** Software, Conceptualization. **Qin Cen:** Methodology, Data curation. **Xuefeng Zeng:** Funding acquisition, Conceptualization. **Yichun Sun:** Project administration, Funding acquisition.

## Declaration of competing interest

The authors declare that they have no known competing financial interests or personal relationships that could have appeared to influence the work reported in this paper.

## Data Availability

Data will be made available on request.

## Appendix A Supplementary data

Supplementary material Image 1
