# Supplementary material for: Innovative research of xian-Ling-Gu-bao herbal residue in regulating soluble carbon-to-nitrogen ratio to promote the growth and nutritional quality of Pleurotus ostreatus: A metabolomics and gut microbiota perspective
Source: Food Chem X. 2025 Dec 29;33:103467. doi: 10.1016/j.fochx.2025.103467 (PMC13216736; doi:10.1016/j.fochx.2025.103467)
Supplement: Supplementary file 1 — Supplementary material [file mmc1.docx]

Supplementary materials


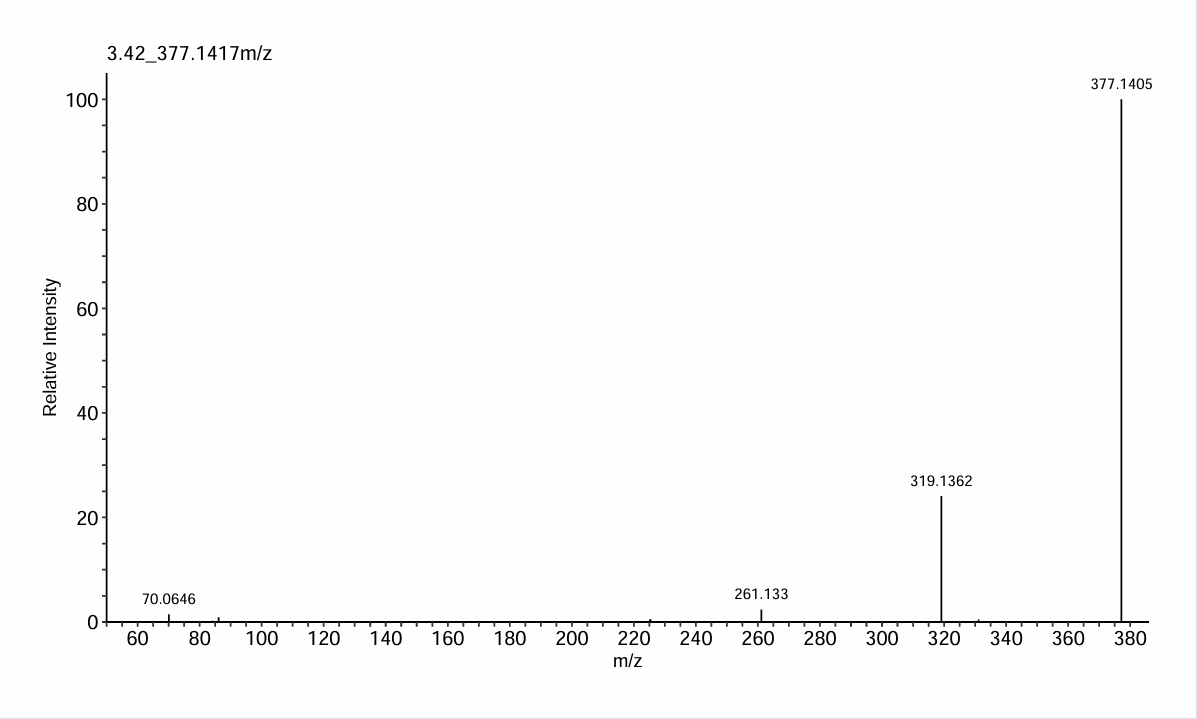


Figure_S1. Secondary mass spectrometry spectra of 8-Epiloganic Acid


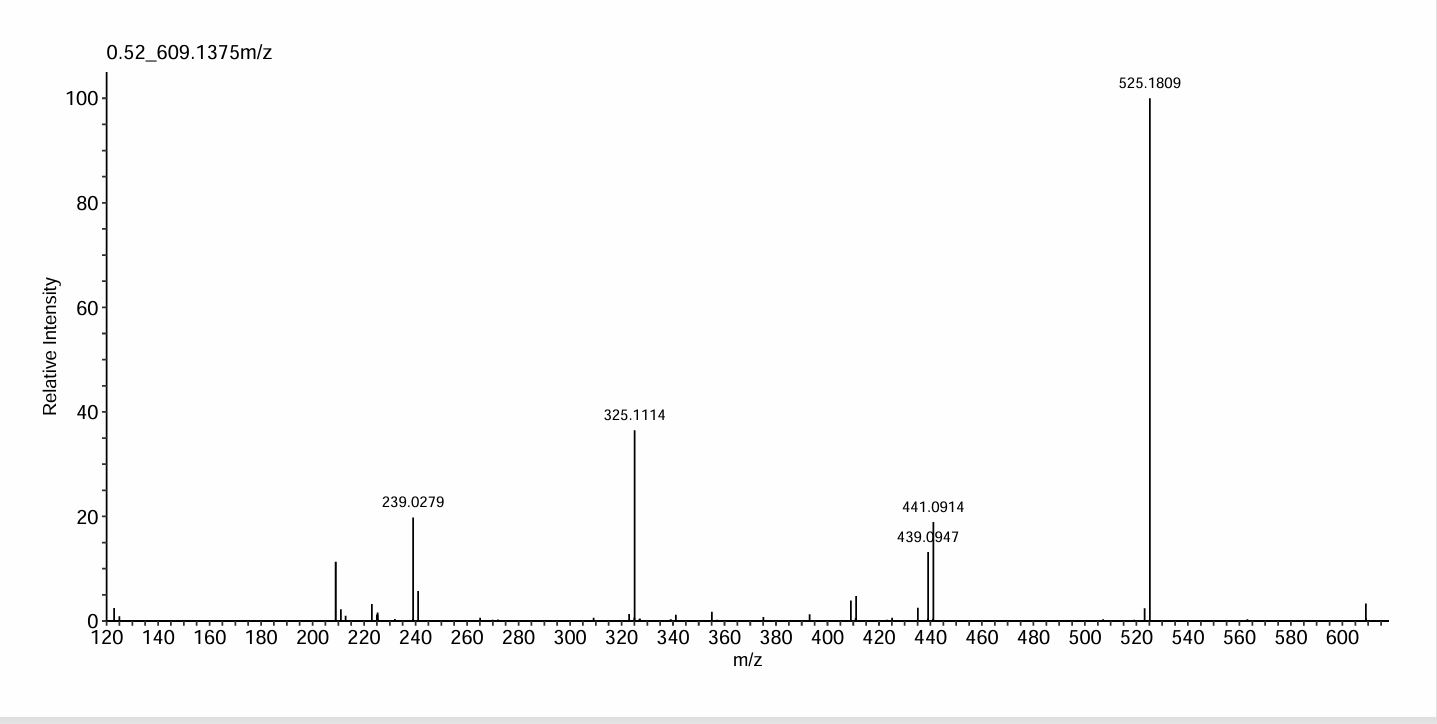


Figure_S2. Secondary mass spectrometry spectra of Apiumoside


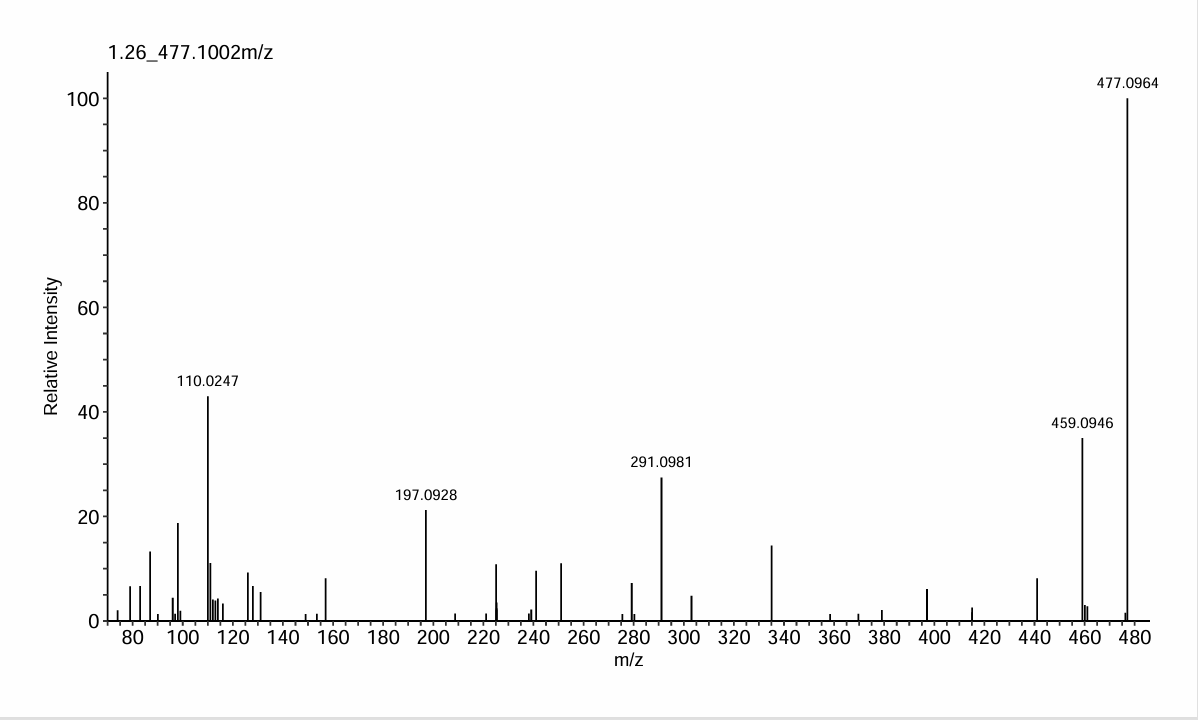


Figure_S3. Secondary mass spectrometry spectra of Cosmosiin


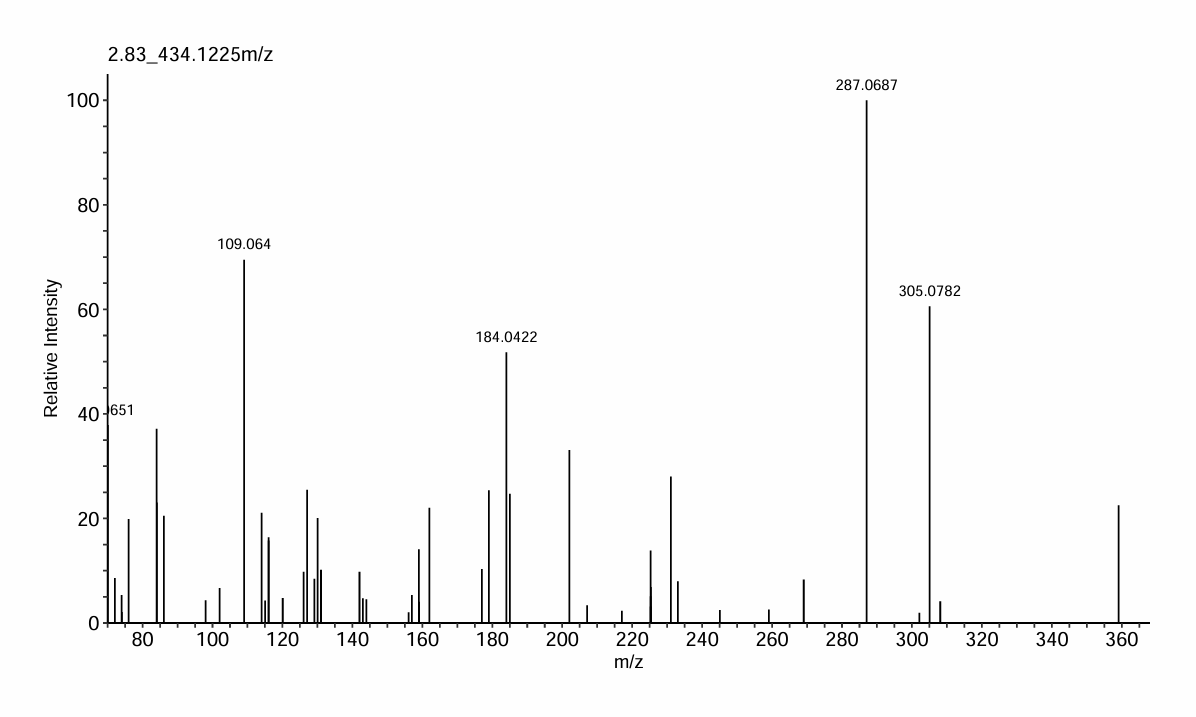


Figure_S4. Secondary mass spectrometry spectra of Engeletin


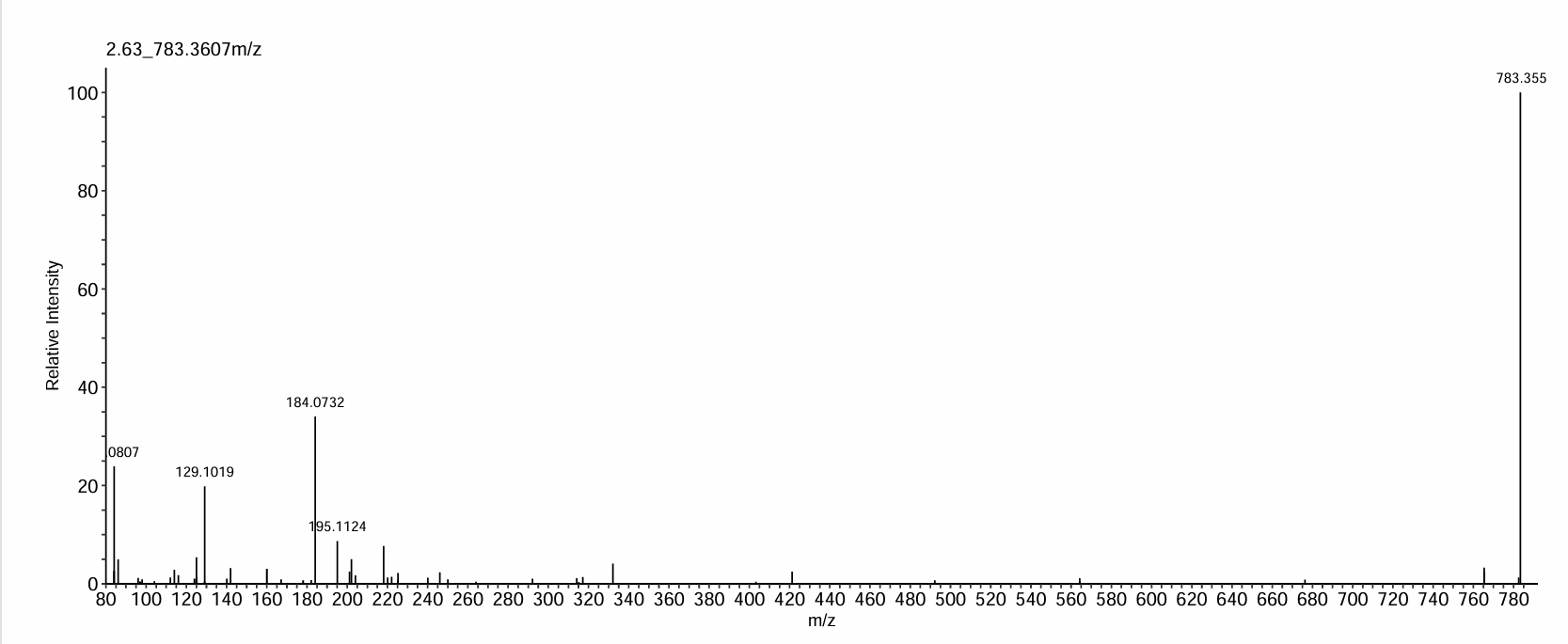


Figure_S5. Secondary mass spectrometry spectra of Flavoxate


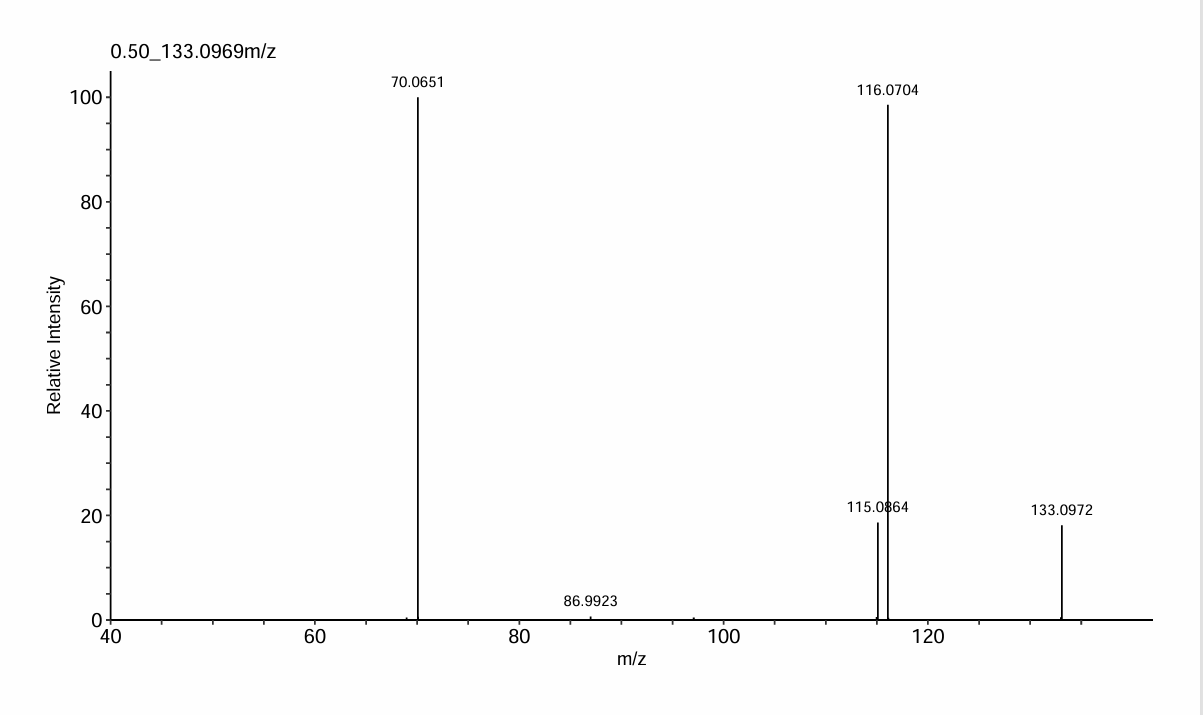


Figure_S6. Secondary mass spectrometry spectra of L-Ornithine


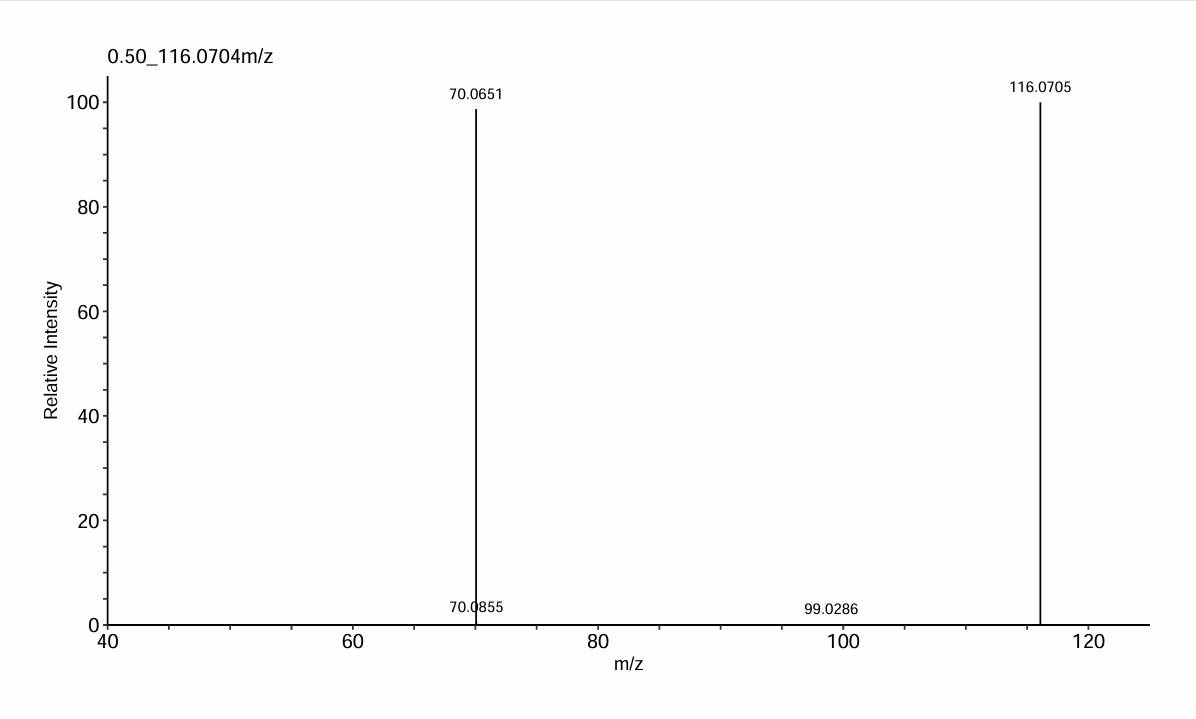


Figure_S7. Secondary mass spectrometry spectra of L-Proline


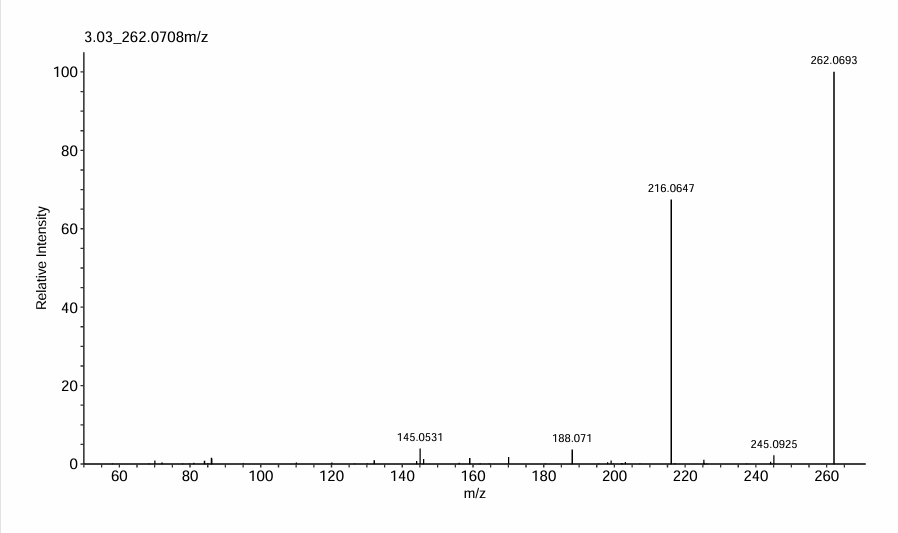


Figure_S8. Secondary mass spectrometry spectra of Purpurogallin


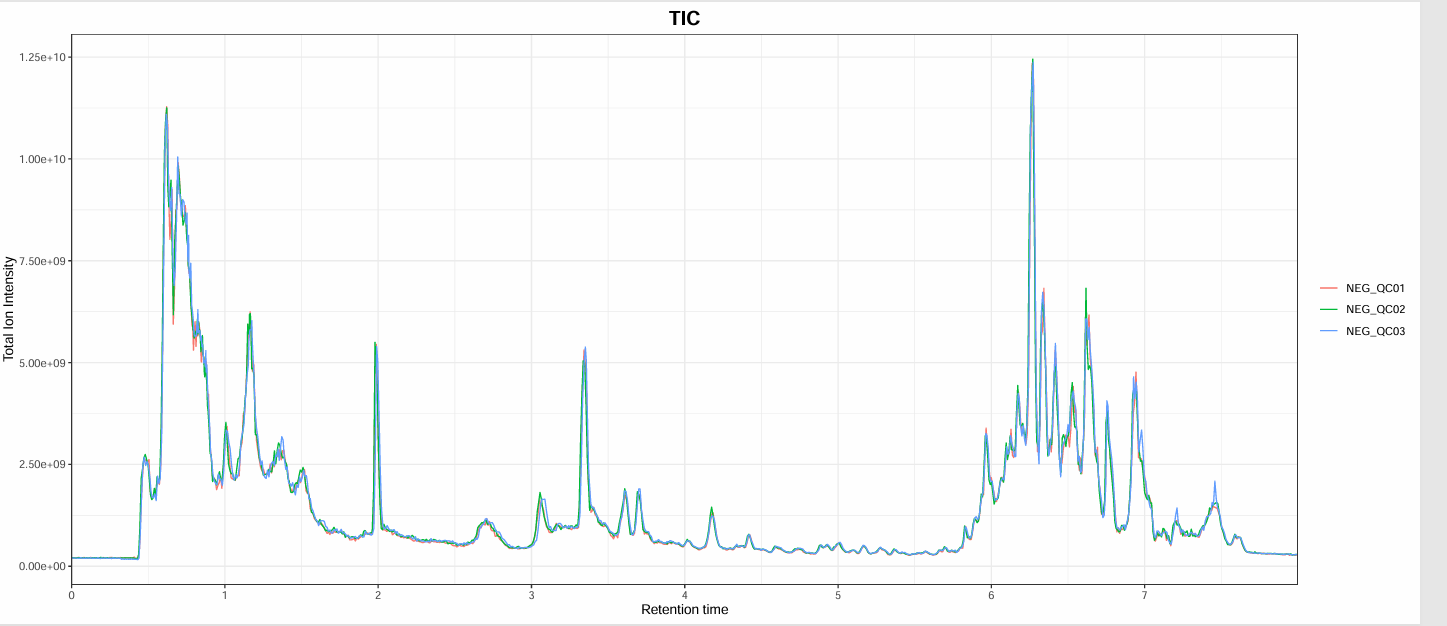


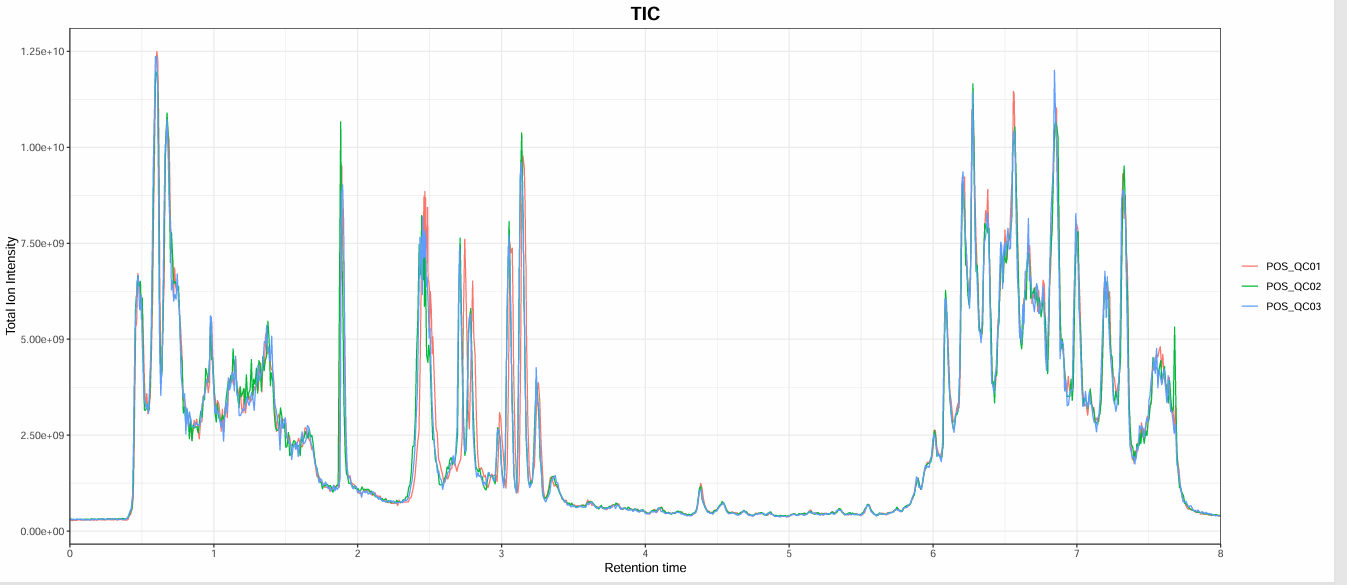


Figure_S9. QC_TIC overlay plot


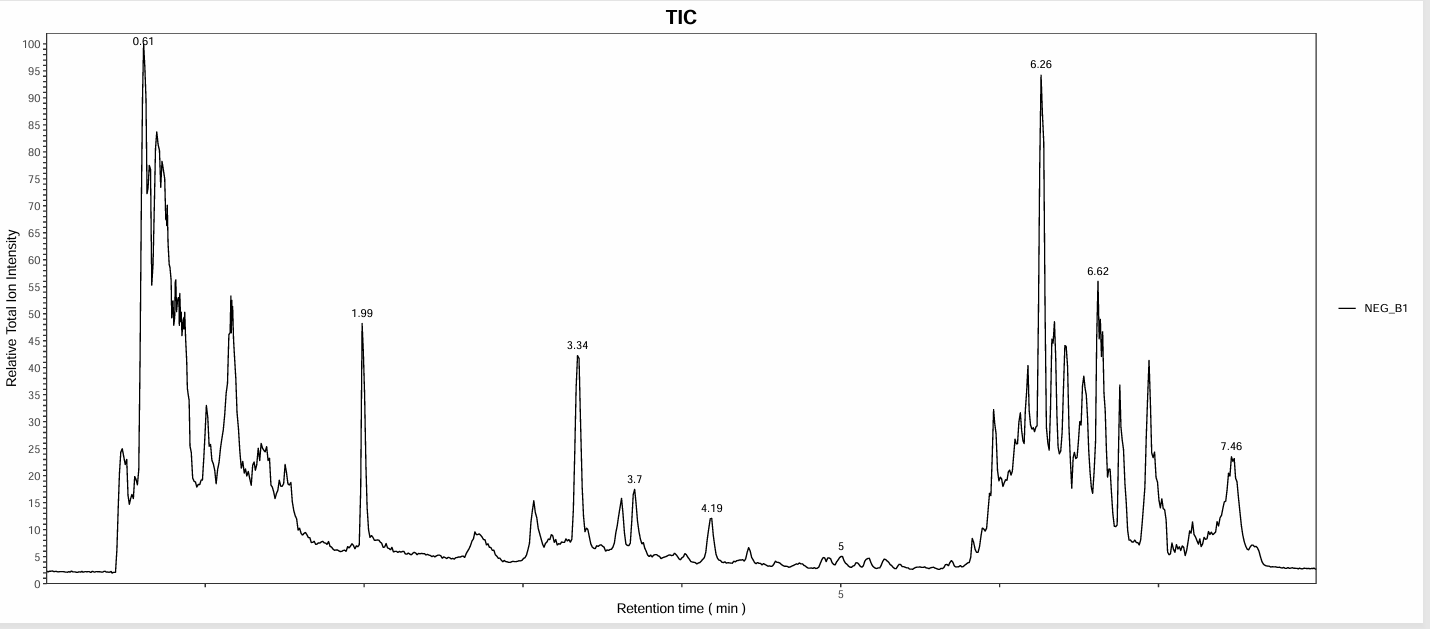


Figure_S10. Total ion chromatogram plot of group 50%CNB.


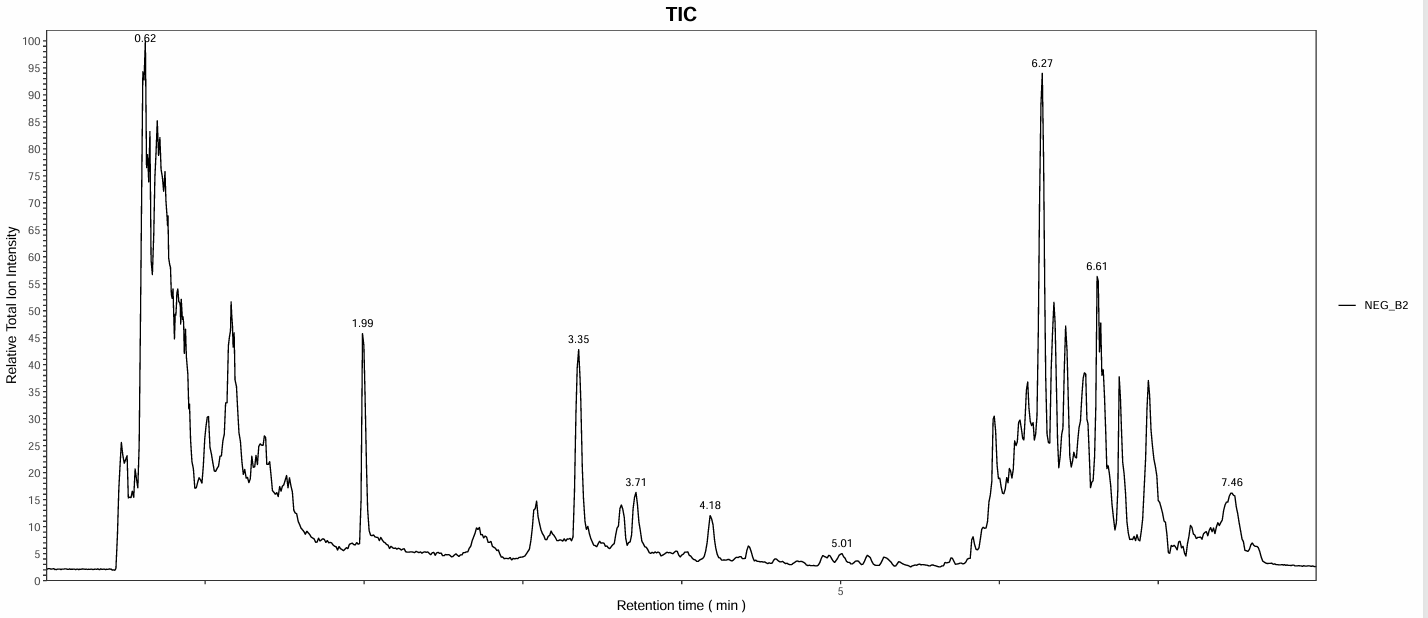


Figure_S11. Total ion chromatogram plot of group 50%CNB.


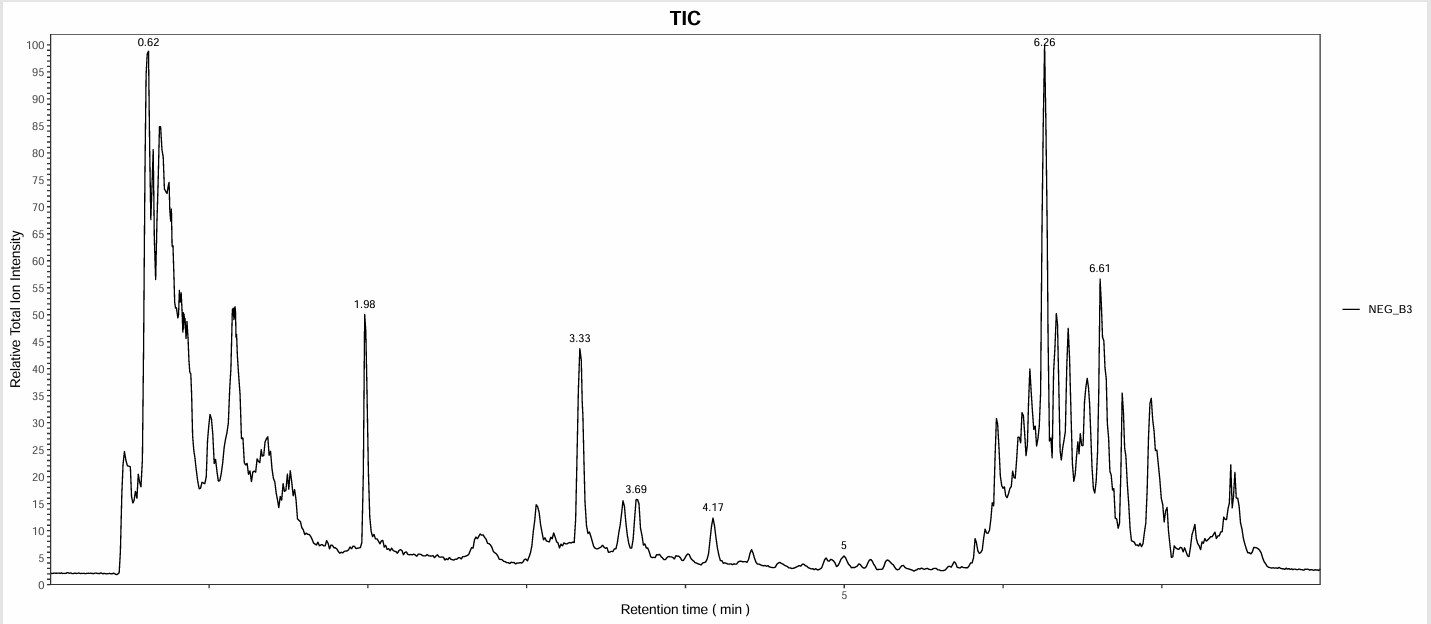


Figure_S12. Total ion chromatogram plot of group 50%CNB.


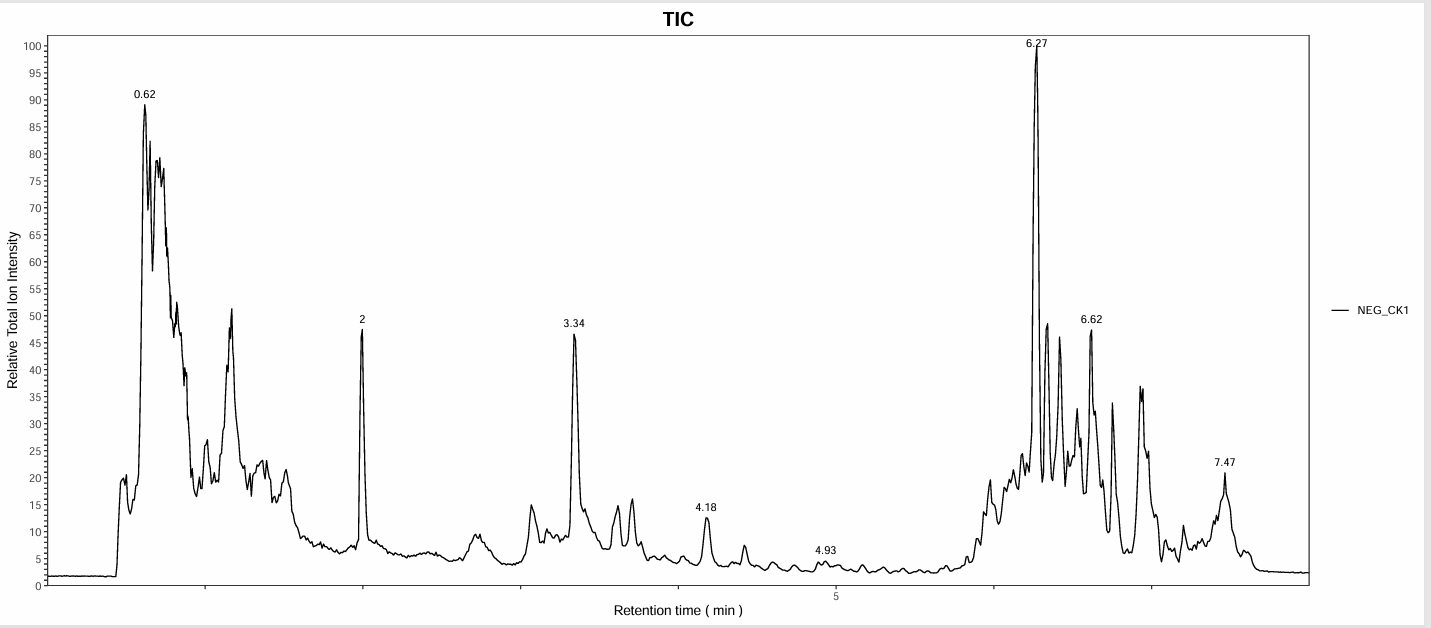


Figure_S13. Total ion chromatogram plot of group 50%XLGB.


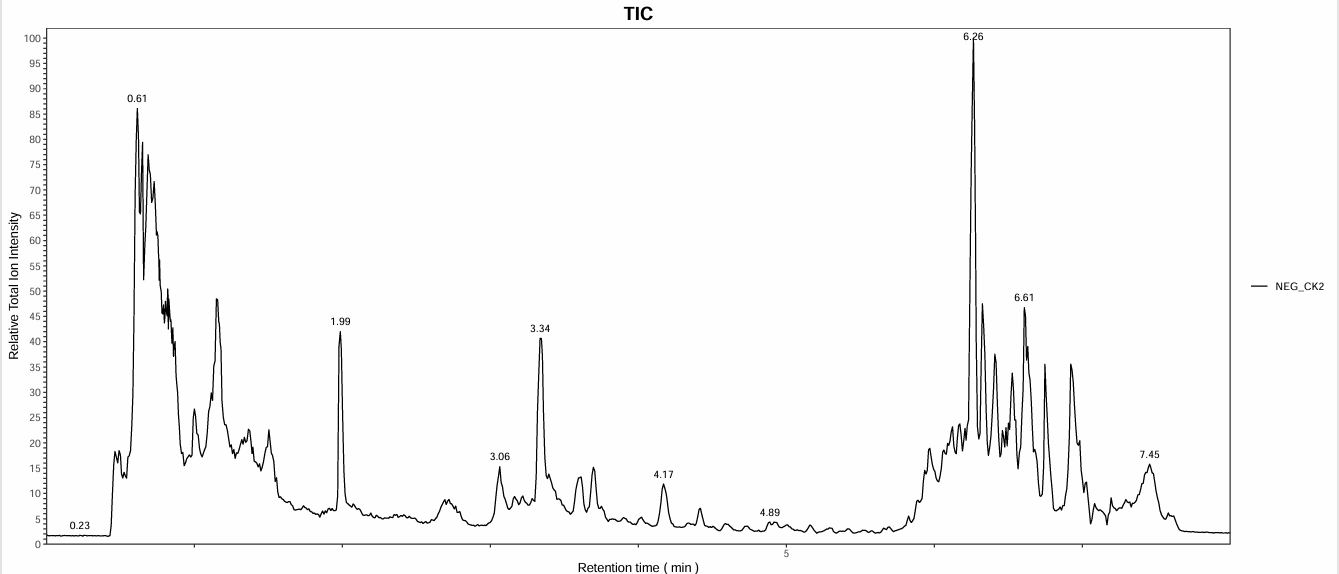


Figure_S14. Total ion chromatogram plot of group 50%XLGB.


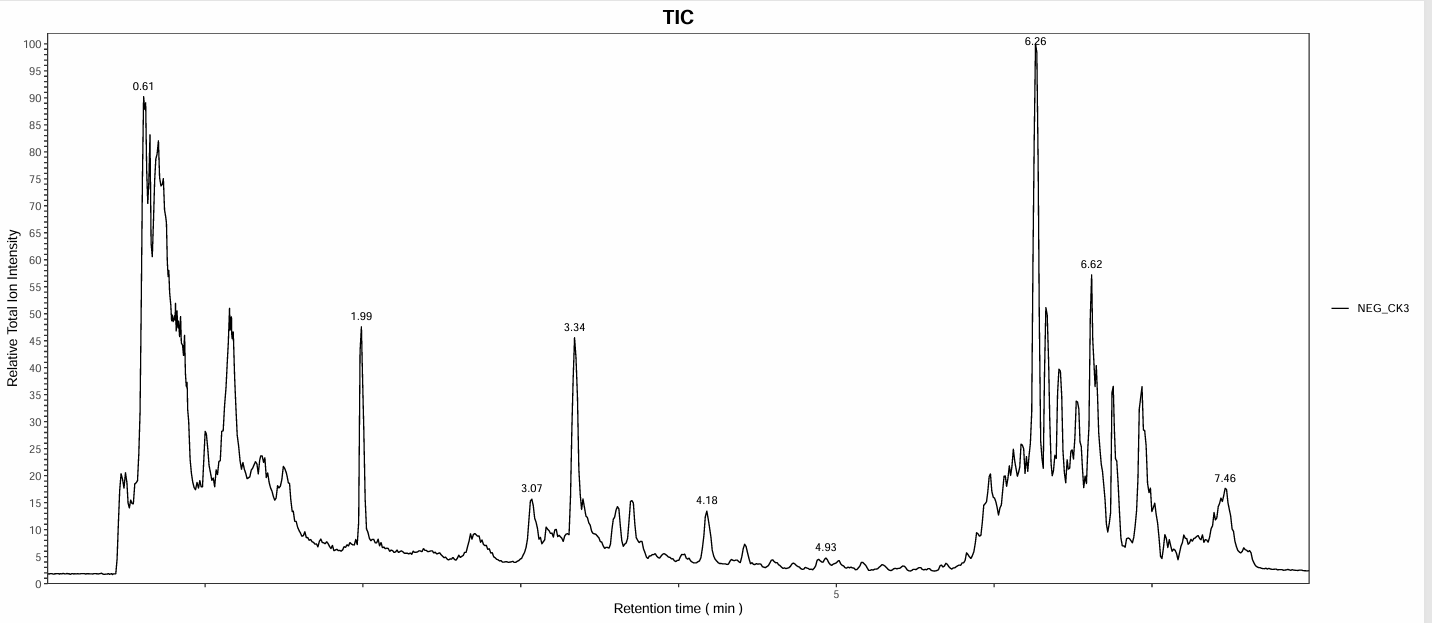


Figure_S15. Total ion chromatogram plot of group 50%XLGB.
